# Investigating biomarkers of mitochondrial and aging-related genes in major depressive disorder through bioinformatics analysis

**DOI:** 10.3389/fpsyt.2025.1653998

**Published:** 2025-09-24

**Authors:** Zhiyuan Chen, Xiaoxiao Tang, Chao Gu, Shaohong Zou

**Affiliations:** Department of Clinical Psychology, People’s Hospital of Xinjiang Uygur Autonomous Region, Urumqi, China

**Keywords:** major depressive disorder, mitochondria-related genes, aging-related genes, biomarker, molecular docking

## Abstract

**Background:**

Major depressive disorder (MDD) is a prevalent mental health condition in which mitochondrial dysfunction and cellular senescence contribute to its pathogenesis. This study aims to identify biomarkers related to mitochondria-associated genes (MRGs) and aging-related genes (ARGs) in MDD using bioinformatics.

**Methods:**

This study utilized data from GSE201332 and GSE52790, including 1,136 MRGs and 866 ARGs. Initially, candidate genes were selected by intersecting MRGs, ARGs, and differentially expressed genes (DEGs) derived from differential expression analysis in GSE201332. Biomarkers were identified through LASSO regression analysis of the candidate genes. The biomarkers were then evaluated using ROC curves, and artificial neural network (ANN) models were constructed. Subsequently, functional enrichment, immune-related analyses, drug predictions, and molecular docking were performed. Finally, the expression of biomarkers was validated using reverse transcription-quantitative polymerase chain reaction (RT-qPCR).

**Results:**

Seven candidate genes were identified from the intersection of 4,041 DEGs, 1,136 MRGs, and 866 ARGs, with SLC25A5, ALDH2, CPT1C, and IMMT identified as potential biomarkers for MDD through LASSO regression analysis. ROC curve analysis in both GSE201332 and GSE52790 showed that these biomarkers effectively distinguished between MDD and control samples, with AUC values exceeding 0.7. ANN models further confirmed the diagnostic potential of these biomarkers. Gene set enrichment analysis (GSEA) revealed significant enrichment of SLC25A5, CPT1C, and IMMT in pathways related to cellular protein complex assembly and chromatin organization. Immune infiltration analysis demonstrated significant positive correlations between SLC25A5, ALDH2, and IMMT and most of the 18 immune cell types. Molecular docking predictions identified ALDH2 and SLC25A5 as potential targets for specific drugs, with NITROGLYCERIN showing the best binding affinity to ALDH2 (-6.4 kcal/mol). RT-qPCR validation showed significantly lower expression of SLC25A5 and IMMT, and higher expression of CPT1C, in patients with MDD compared to controls (p < 0.05), consistent with bioinformatics predictions.

**Conclusion:**

This study identified SLC25A5, ALDH2, CPT1C, and IMMT as biomarkers associated with MDD, offering insights into its molecular mechanisms.

## 1 Introduction

Major depressive disorder (MDD) is a prevalent mental health condition characterized by persistent feelings of sadness, hopelessness, and a loss of interest or pleasure in daily activities. Affecting millions globally, MDD has an estimated lifetime prevalence of around 16.6% in adults, representing a significant public health issue (1). The etiology of MDD is complex, involving a combination of genetic, environmental, and psychological factors that contribute to its onset (2). Its clinical presentation is highly variable, encompassing emotional disturbances, cognitive impairments, and somatic symptoms, all of which can severely affect an individual’s quality of life and functional capacity (3). Recent epidemiological studies have revealed that MDD disproportionately impacts certain groups, including women, individuals with a family history of depression, and those exposed to chronic stress or traumatic events (4). Despite advancements in understanding the core processes of MDD, the precise mechanisms remain elusive, complicating both diagnosis and treatment (5).

The treatment of MDD includes pharmacotherapy, psychotherapy, and lifestyle changes. However, these approaches are often hindered by challenges such as delayed therapeutic onset, limited response rates, and side effects (3). These obstacles underscore the urgent need for novel therapeutic strategies and biomarkers to facilitate earlier diagnosis and more effective management of MDD. Identifying new biological markers and elucidating the molecular mechanisms underlying MDD may offer critical insights into its pathophysiology, potentially leading to improved diagnostic and therapeutic approaches (2).

Mitochondria, the powerhouse of the cell, play a pivotal role not only in energy production but also in regulating essential processes such as apoptosis (programmed cell death), calcium homeostasis, and cellular metabolism (6). Disruption of mitochondrial function has been implicated in various diseases, including neurodegenerative disorders and metabolic syndromes, suggesting that such dysfunctions may contribute significantly to the pathophysiology of MDD (7). Mitochondrial dysfunction in aging is marked by increased oxidative stress, reduced bioenergetics, and impaired mitochondrial dynamics, which may promote the onset and progression of depressive symptoms (8). Recent studies have indicated that mitochondrial dysfunction and age-related pathways are closely linked to MDD development, although the exact molecular mechanisms remain unclear (9).

This study utilizes bioinformatics techniques to analyze transcriptomic data from patients with MDD and healthy controls obtained from the GEO database. Differential expression analysis is employed to identify mitochondrial and aging-related biomarkers associated with MDD. Additionally, an artificial neural network (ANN) model is constructed to evaluate the diagnostic potential of these biomarkers. Through functional enrichment analysis, immune cell infiltration studies, and molecular docking, the study aims to elucidate the complex roles of these biomarkers in MDD pathogenesis, highlighting their therapeutic potential.

Exploring the relationship between mitochondrial function, aging, and MDD is crucial for enhancing our understanding of the disorder. By investigating the molecular foundations of these interactions, the goal is to provide valuable insights into MDD pathophysiology and identify potential biomarkers that could improve clinical management and treatment outcomes.

## 2 Materials and methods

### 2.1 Data sources

**Figure 1:**
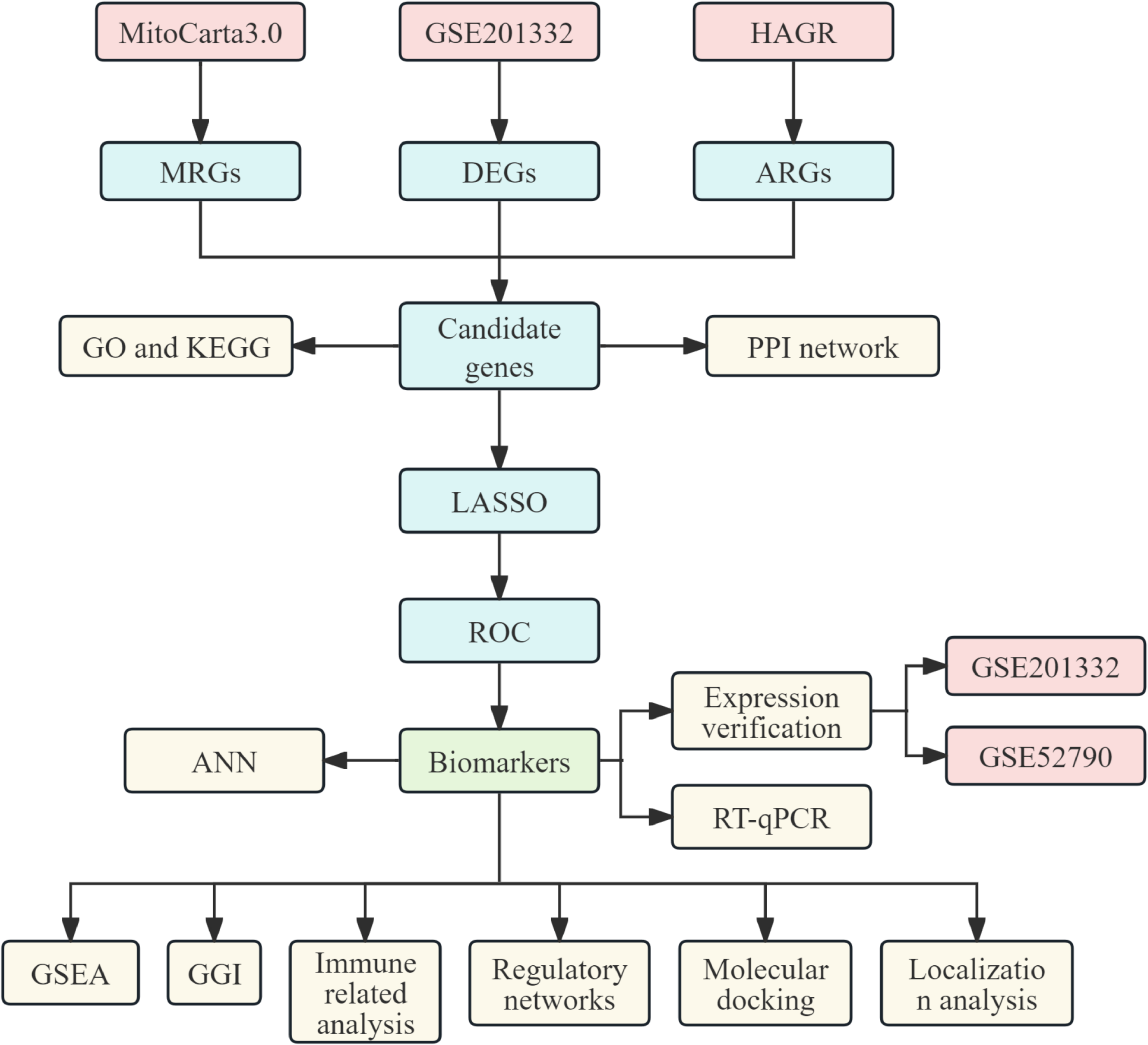
Flowchart of the research process for biomarker screening.

This study utilized MDD-related datasets obtained from GEO (http://www.ncbi.nlm.nih.gov/geo/). The GSE201332 dataset, serving as the training set, included whole blood samples from 20 patients with MDD and 20 healthy controls (**Supplementary Table 1**), while GSE52790, used as the validation set, comprised whole blood samples from 10 patients with MDD and 12 healthy controls. MitoCarta3.0 (https://www.broadinstitute.org/mitocarta) and the HAGR database (https://genomics.senescence.info/) were used to extract 1,136 mitochondria-related genes (MRGs) and 866 aging-related genes (ARGs), respectively. A flowchart of the study is provided in **Figure 1**.

### 2.2 Differential expression analysis and functional enrichment analysis

Differential expression analysis was performed to identify disease-related genes by screening for differentially expressed genes (DEGs) between the MDD and control groups using the Limma package (v 3.44.3) in GSE201332, applying thresholds of an adjusted p-value < 0.05 and |Log_2_FC| > 0.5 (10). The intersection of MRGs, ARGs, and DEGs was then analyzed to pinpoint potential biomarkers. GO and KEGG functional enrichment analyses were conducted using the clusterProfiler package (v 4.0.2) (p-value < 0.05, count > 1) (11). The candidate genes were further analyzed using the STRING database to construct a protein-protein interaction (PPI) network with a threshold of 0.2.

### 2.3 Identification of biomarkers and construction of ANN

For biomarker screening, LASSO logistic regression was performed on the candidate genes using the glmnet package (v 4.0-2) (12), with family = “binomial” to accommodate the binary outcome variable. To ensure reproducibility, set.seed (30) was used to fix random number generation, and a maximum of 5,000 iterations was allowed to ensure algorithm convergence. Ten-fold cross-validation (nfolds = 10) was employed to evaluate model performance and determine the optimal regularization strength (λ). The λ value with the smallest cross-validation error (lambda.min) was chosen for model fitting, and genes with non-zero coefficients were identified as potential biomarkers. The diagnostic performance of these biomarkers was further evaluated through Receiver Operating Characteristic (ROC) curves in both GSE201332 and GSE52790. The ROC curve plotted the false positive rate on the x-axis and the true positive rate on the y-axis, with the Area Under the Curve (AUC) serving as the quantitative measure. An AUC of 0.5 indicated random guessing, an AUC > 0.7 indicated good discriminative ability, and an AUC > 0.9 suggested excellent diagnostic performance. Genes with an AUC > 0.7 were selected for further analysis. Finally, an ANN model based on the biomarkers was developed using the neuralnet package to further assess their diagnostic performance. The ROC curves for the ANN models were also evaluated in both datasets.

### 2.4 Functional analysis of biomarkers

To elucidate the regulatory mechanisms and biological functions of biomarkers, gene set enrichment analysis (GSEA) was performed using the clusterProfiler package (v 4.0.2) (11). GO gene sets were sourced from the org.Hs.eg.db database *via* the gseGO function, and KEGG gene sets were retrieved using the gseKEGG function with the organism parameter set to “hsa”. First, correlation coefficients between each biomarker and the expression levels of all genes were calculated, and genes were ranked based on these coefficients, from high to low. Enrichment scores were determined using the classic permutation method of GSEA (gene set permutation), with significantly enriched gene sets identified using a threshold of p-value < 0.05. In addition, GeneMANIA (https://genemania.org/) was used to predict the genes and functions associated with the biomarkers.

### 2.5 Immune-related analyses

To explore immune cell-related variations, the study investigated the differences in ssGSEA scores for 29 immune cell types between individuals with neurodegenerative diseases (NDD) and control subjects in the GSE201332 dataset (13). The associations between differentially expressed immune cells and the biomarkers were then analyzed. Moreover, to assess the relationship between biomarkers and immunological factors, various immunomodulators and chemokines were retrieved from the ISIDB database (http://cis.hku.hk/TISIDB/), and their correlations with biomarkers were evaluated using Spearman’s method (|cor| > 0.3, p-value < 0.05).

### 2.6 Regulatory network analysis

To examine the molecular regulatory mechanisms of the biomarkers, the NetworkAnalyst platform was used to access the “CORE vertebrates” dataset from the JASPAR database (https://www.networkanalyst.ca/). Transcription factors (TFs) with potential binding affinity to the biomarkers were identified using a motif matching score ≥ 800 and a corresponding p-value ≤ 1e-4. miRNAs related to the biomarkers were then predicted using the miRWalk 3.0 (http://mirwalk.umm.uni-heidelberg.de/) and Starbase v3.0 (http://starbase.sysu.edu.cn/) databases, with Starbase requiring a “Pan-Cancer Conservation” score ≥ 3. The miRNAs identified from both databases were intersected to determine the target miRNAs. Next, lncRNAs corresponding to these miRNAs were predicted using the miRNet 2.0 database (https://www.mirnet.ca/), with the screening condition set as CancerNum > 0 in Starbase v3.0. The lncRNAs identified by both databases were intersected to establish the final set of target lncRNAs. Finally, a TF-gene and lncRNA-miRNA-mRNA regulatory network was constructed using Cytoscape software to visually depict these complex molecular interactions.

### 2.7 Drug prediction and molecular docking

To assess the effects of chemotherapeutic drugs on biomarkers, a drug-gene network was constructed using the DSigDB database (https://dsigdb.tanlab.org/DSigDBv1.0/). The structural information for the chemotherapeutic drugs in this network was sourced from PubChem (https://pubchem.ncbi.nlm.nih.gov/). Concurrently, protein sequences and functional data for the key genes were retrieved from the Uniprot database (https://www.uniprot.org/), and their three-dimensional structures were obtained from the PDB database (https://www.rcsb.org/). These protein and drug structures were uploaded to the CB-Dock2 platform (https://cadd.labshare.cn/cb-dock2/php/index.php) for molecular docking analysis. The platform standardized protonation states and automatically identified and defined binding sites using its built-in algorithm. Binding affinity between the proteins and ligands was evaluated based on binding energy, with lower values indicating stronger binding. To validate the stability and reliability of the docking results, additional docking experiments were performed using alternative receptor structures of known binders to assess the binding interactions.

### 2.8 Drug prediction and molecular docking

To further elucidate the mechanism of action of drugs and evaluate the stability of drug-biomarker complexes as well as the kinetic characteristics of drug binding, molecular dynamics simulations were conducted using GROMACS 2024.4 software. The simulations followed the AMBER99SB-ILDN force field and utilized the TIP3P water model. A cubic system box was set, ensuring the box edges were 1 nm away from the protein edges, and 0.15 mol/L Na^+^/Cl⁻ ions were added to maintain electrical neutrality. Energy minimization was first performed using the steepest descent method. Subsequently, both a heat bath (NVT, with fixed particles, volume, and temperature) and a pressure bath (NPT, with fixed particles, pressure, and temperature) were applied, employing the V-rescale method for temperature coupling. The reference temperature was set to 300 K, with a time step of 2 femtoseconds, and each simulation phase lasted 100 picoseconds. The final molecular dynamics simulation ran for 20 nanoseconds. To quantify binding characteristics, the root-mean-square deviation (RMSD) of backbone atoms in the protein-ligand complex was calculated to assess conformational stability. The root-mean-square fluctuation (RMSF) of protein backbone atoms was analyzed to observe changes in residue flexibility, and fluctuations in total system energy were monitored to evaluate thermodynamic stability. Additionally, the number of hydrogen bonds and their occupancy between the drug and target were counted to quantify the strength of binding interactions. The distance between the small molecule binding site and the amino acid residues of the protein was measured to evaluate binding stability, interaction mechanisms, and conformational changes.

### 2.9 Subcellular localization, chromosomal localization, and association analysis with disease risk of biomarkers

The position of the biomarker on the chromosome was visualized using the RCircos package (v 1.2.2) (14). Gene sequences for the biomarkers were retrieved from the NCBI database, and subcellular localization was assessed using the mRNALocator database. The relationship between the biomarkers and MDD risk was explored using the CTD database.

### 2.10 Biomarker expression analysis

To further validate biomarker expression in MDD and control groups, expression levels were analyzed in both the training and validation sets, followed by RT-qPCR validation. Five pairs of whole blood samples were obtained from patients with MDD and healthy controls (**Supplementary Table 2**) at the People’s Hospital of Xinjiang Uygur Autonomous Region for qRT-PCR analysis.

The study cohort consisted of female patients aged 40 to 50 years, diagnosed with depression, who sought care at the Department of Clinical Psychology, People’s Hospital of Xinjiang Uygur Autonomous Region, in June 2024. A control group of healthy females in the same age range was also included. Inclusion criteria were as follows: (1) Diagnosis: Participants met DSM-5 criteria for MDD, confirmed through structured clinical interviews; (2) Symptom severity: A baseline score ≥18 on the 17-item Hamilton Depression Rating Scale (HAMD-17), indicating moderate-to-severe depression; (3) Age: Adults aged 18–65 years; (4) Treatment status: Participants were not receiving any medication or psychological treatment at the time of enrollment. Exclusion criteria were as follows: (1) Comorbid psychiatric disorders: Axis I disorders (e.g., bipolar disorder, psychosis, primary anxiety disorders) or substance use disorders (within 6 months); (2) High suicide risk: Defined by HAMD item 3 score ≥3, recent suicide attempt, or active ideation with intent; (3) Unstable medical conditions (e.g., neurological disorders, uncontrolled diabetes) or medications influencing mood (e.g., corticosteroids). Participants were required to fast overnight for at least 8 hours before blood collection, which was performed between 8:00 AM and 10:00 AM under controlled temperature and lighting conditions. Blood was drawn by a trained phlebotomist using sterile techniques, and aliquots were stored at -20°C for no more than 2 weeks.

This study was approved by the Clinical Research Ethics Committee of the People’s Hospital of Xinjiang Uygur Autonomous Region (KY2024070801), and all patients provided signed informed consent. To validate biomarker expression, total RNA was extracted from the samples using TRIZOL, according to the manufacturer’s instructions. The first strand of complementary DNA (cDNA) was synthesized from 2 μg of total RNA using the SureScript First Strand cDNA Synthesis Kit (Servicebio, Wuhan, China). RT-qPCR was performed with the 2xUniversal Blue SYBR Green qPCR Master Mix (Servicebio, Wuhan, China). The reaction protocol was as follows: 1 minute at 95°C, followed by 40 cycles of 20 seconds at 95°C, 20 seconds at 55°C, and 30 seconds at 72°C. Primer sequences are listed in **Table 1** and were validated for specificity using BLAST. GAPDH was used as the internal reference gene. Gene expression levels were calculated using the 2^-△△Ct^ method (15). Data analysis and visualization were performed using GraphPad Prism 5 (GraphPad Software Inc., USA).

**Table 1:** Primer sequences.

Primers	Sequences (5’-3’)	Amplification size (bp)
CPT1C-F	GGCTAGGGACACGAGAGAGA	112
CPT1C-R	CCAATCCCAGTGCAAGGAGT	
SLC25A5-F	AGACTGCGTGGTCCGTATTC	190
SLC25A5-R	TGCCAGATTCCCTGCAAAGT	
ALDH2-F	GCATGGACGCATCACACAG	103
ALDH2-R	TTGCCATTGTCCAGGGTCTC	
IMMT-F	CACCTACAGAAGCGGCTCAA	139
IMMT-R	TCTGAAAGTGCAGGTGTGGG	
GAPDH-F	CGAAGGTGGAGTCAACGGATTT	148
GAPDH-R	ATGGGTGGAATCATATTGGAAC	

### 2.11 Statistical analysis

Bioinformatics analysis was performed using R software. Statistical significance was set at p < 0.05. Due to the small sample size, non-parametric tests (Mann–Whitney U test) were used for group comparisons in PCR experiments to ensure the robustness of the results.

## 3 Results

### 3.1 A total of 7 candidate genes were subjected to functional enrichment analysis

**Figure 2:**
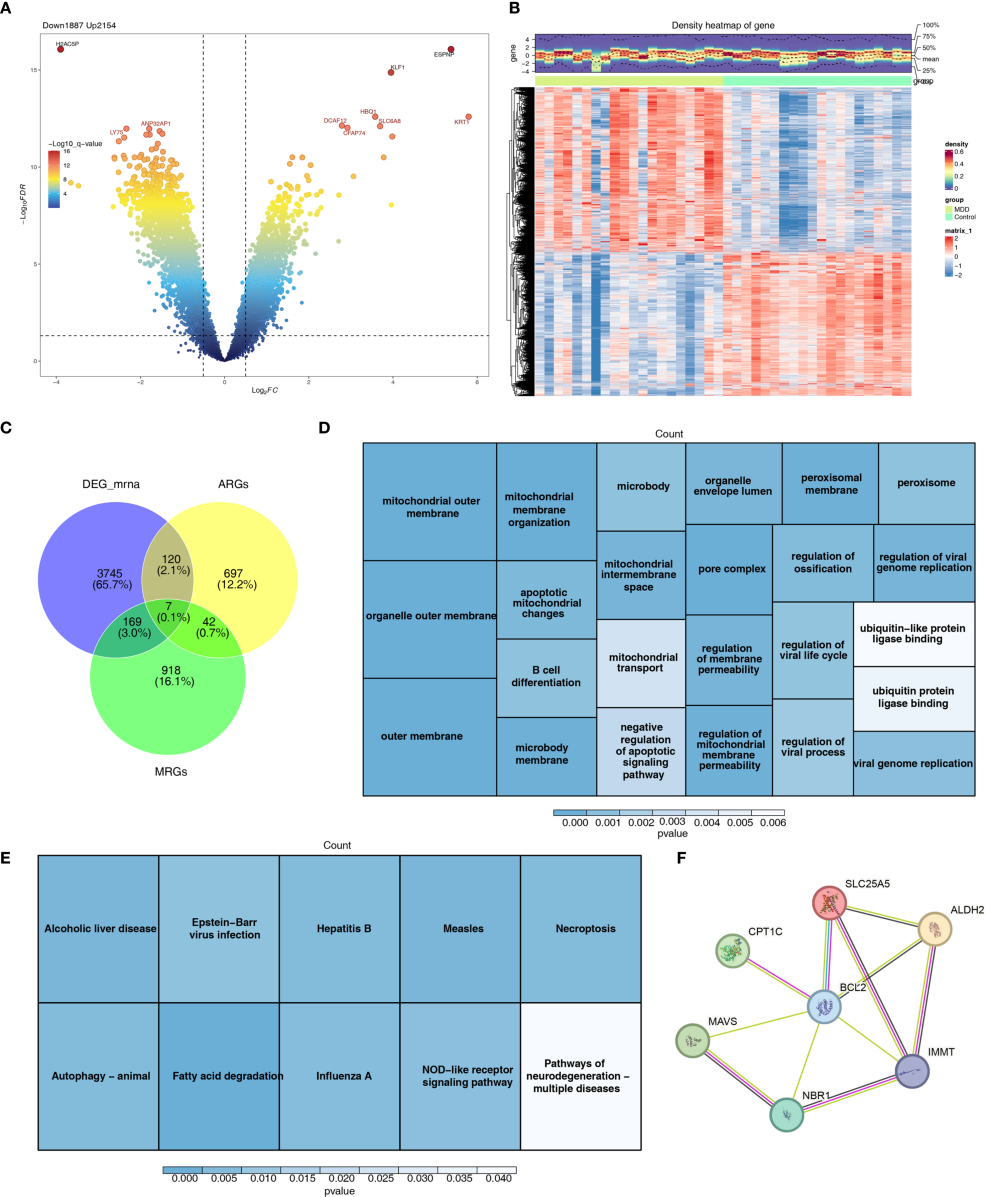
Differential expression analysis and functional enrichment analysis. **(A)** Volcano plot of differentially expressed genes between MDD and control groups. **(B)** Heat map of differentially expressed genes between MDD and control groups. **(C)** Venn diagram identifying candidate genes. **(D)** GO enrichment analysis results of candidate genes. **(E)** KEGG enrichment analysis results of candidate genes. **(F)** Protein interaction network of candidate genes.

A total of 4,041 DEGs were identified in GSE201332, comprising 2,154 upregulated genes and 1,887 downregulated genes (**Figures 2A, B**). Seven candidate genes were further selected by intersecting DEGs with 1,136 MRGs and 866 ARGs (**Figure 2C**). GO enrichment analysis revealed that these candidate genes were associated with 47 functional categories, including mitochondrial outer membrane, organelle outer membrane, and other relevant terms (**Figure 2D**). KEGG pathway analysis highlighted the involvement of these genes in 10 signaling pathways, such as fatty acid degradation and NOD-like receptor signaling (**Figure 2E**). The PPI network analysis identified key PPIs, including MAVS-BCL2 and NBR1-MMT (**Figure 2F**).

### 3.2 SLC25A5, ALDH2, CPT1C, and IMMT had excellent diagnostic performance for MDD

**Figure 3:**
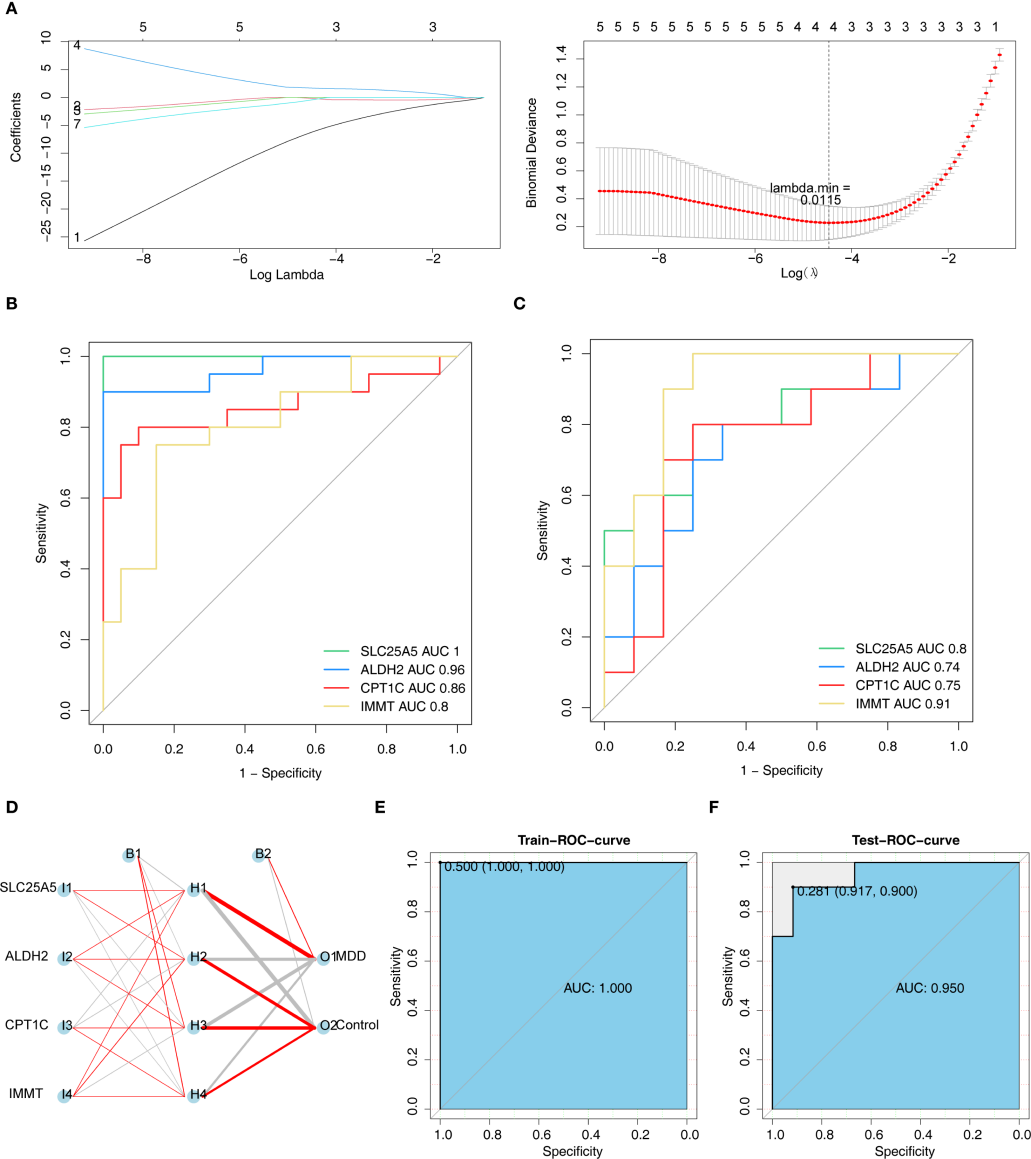
SLC25A5, ALDH2, CPT1C, and IMMT had excellent diagnostic performance for MDD. **(A)** LASSO regression analysis was used to screen biomarkers. The left panel depicted the coefficient trajectory plot of genes. The horizontal axis represented the logarithm of the regularization parameter λ (Log Lambdas), and the vertical axis denoted the regression coefficients of genes. Lines of different colors corresponded to the coefficient trajectories of candidate genes as λ varied: blue for SLC25A5, red for ALDH2, yellow for CPT1C, and gray for IMMT. The right panel showed the cross - validation error curve. The shaded area indicated the standard error of the error, and the red curve represented the binomial deviance. **(B, C)** ROC curve analysis of biomarkers (GSE201332 training set and GSE52790 validation set). The horizontal axis stood for 1−Specificity, and the vertical axis represented Sensitivity. **(D)** Artificial neural network diagnostic model constructed based on biomarkers. Red-colored connections indicated positive corresponding weights, while gray - colored ones indicated negative weights. **(E)** ROC was used to evaluate the performance of the artificial neural network in the training set and validation set.

In LASSO regression analysis, the lowest error during cross-validation was achieved with a lambda.min of 0.0115, which led to the selection of four biomarkers: SLC25A5, ALDH2, CPT1C, and IMMT (**Figure 3A**). The ROC curve analysis showed AUC values greater than 0.7 in both GSE201332 and GSE52790, indicating that these biomarkers could effectively differentiate between MDD and control samples (**Figures 3B, C**). The ANN model built using these biomarkers demonstrated excellent diagnostic performance in both the training and validation sets, with AUC values of 1 and 0.95, respectively (**Figures 3D, E**).

### 3.3 Biomarkers had different biological functions

**Figure 4:**
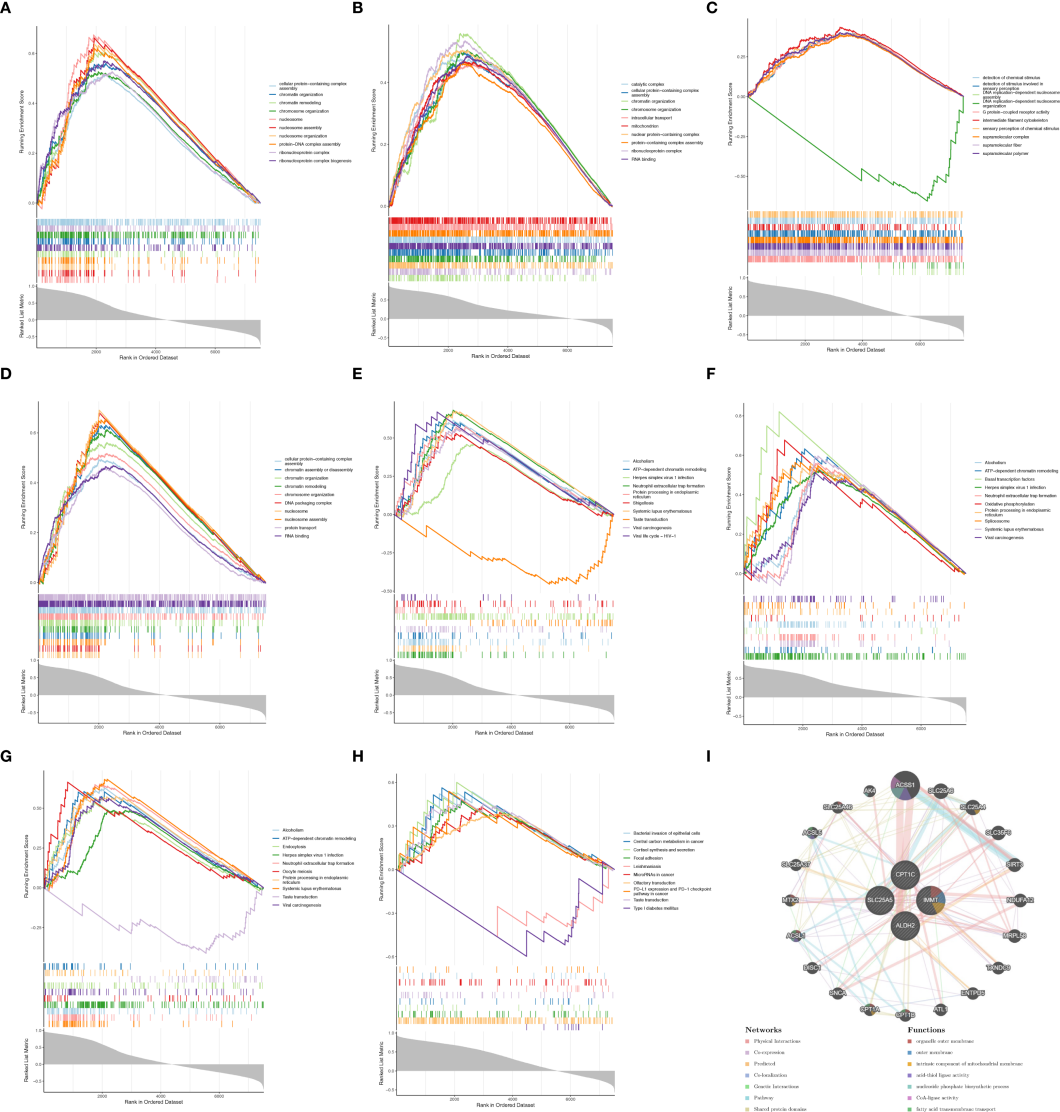
Biomarkers had different biological functions. GO enrichment analysis results for **(A)** SLC25A5, **(B)** CPT1C, **(C)** IMMT, and **(D)** CPT1C. KEGG enrichment analysis results for **(E)** SLC25A5, **(F)** CPT1C, **(G)** IMMT, and **(H)** CPT1C. **(I)** Gene-gene interaction of biomarkers.

GO enrichment analysis indicated that SLC25A5, CPT1C, and IMMT were significantly associated with processes such as cellular protein-containing complex assembly, chromatin organization, and chromosome organization (**Figures 4A–C**). Additionally, CPT1C was linked to the detection of chemical stimuli and sensory perception (**Figure 4D**). In KEGG pathway analysis, SLC25A5, CPT1C, and IMMT were implicated in various biological processes, including ATP-dependent chromatin remodeling, neutrophil extracellular trap formation, and protein processing in the endoplasmic reticulum (**Figures 4E–G**). Moreover, CPT1C was involved in pathways such as cortisol synthesis and secretion, and focal adhesion (**Figure 4H**). The gene-gene interaction (GGI) network revealed additional genes related to biomarkers, including ACSS1 and SLC25A6, which are involved in functions such as organelle outer membrane composition, fatty acid transmembrane transport, and other processes (**Figure 4I**). GO and KEGG pathway enrichment analysis results for the four biomarkers are provided in **Supplementary Table 3**.

### 3.4 Biomarkers correlated with both different immune cells and immune factors

**Figure 5:**
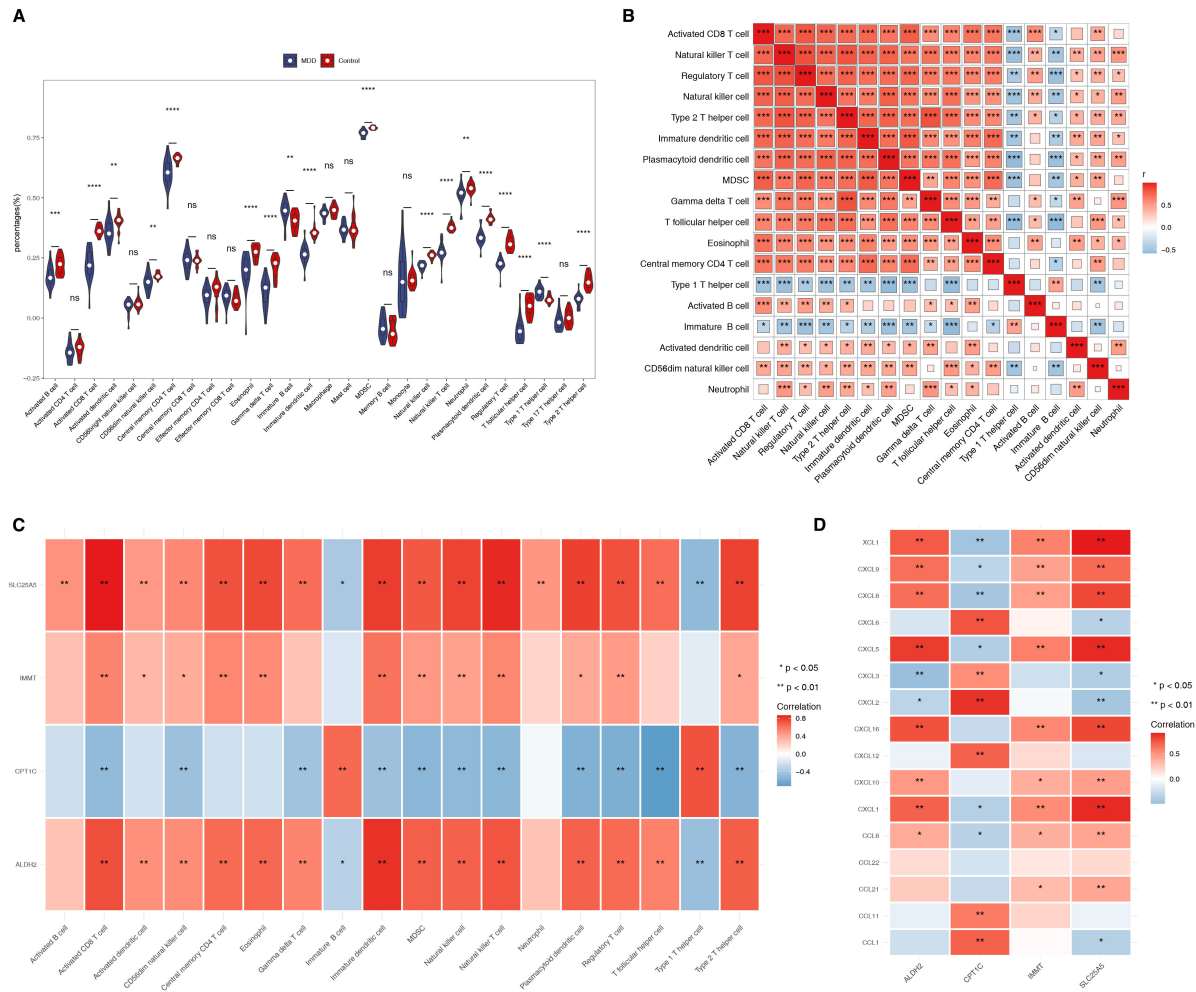
Biomarkers correlate with both different immune cells and immune factors. **(A)** The ssGSEA algorithm revealed substantial differences in the scores of 18 immune cells between the MDD and control groups. ns represented no significance, ****p-value<0.0001. **(B)** Heat map showing the correlation analysis of differential immune cells. *p-value<0.05, **p-value<0.01, ***p-value<0.001. **(C)** Correlation analysis between differential immune cells and biomarkers. **(D)** Heat map of correlation between biomarkers and immune factors.

The ssGSEA algorithm revealed significant differences in the scores of 18 immune cells between the MDD and control groups. For example, activated B cells, activated CD8 T cells, and activated dendritic cells (DCs) displayed reduced expression levels in the MDD group (**Figure 5A**). Most of these immune cell types were positively correlated with one another (**Figure 5B**). Additionally, SLC25A5, ALDH2, and IMMT showed positive correlations with several differential immune cells, including activated B cells, activated CD8 T cells, and activated DCs. In contrast, CPT1C exhibited an inverse relationship with most immune cells, except for Immature B cells and Type 1 T helper cells (**Figure 5C**). Correlation analysis with immune factors demonstrated significant associations between the biomarkers and XCL1, CXCL9, CXCL8, CXCL5, CXCL1, and CCL8 (**Figure 5D**).

### 3.5 Regulatory networks and molecular docking of biomarkers were performed

**Figure 6:**
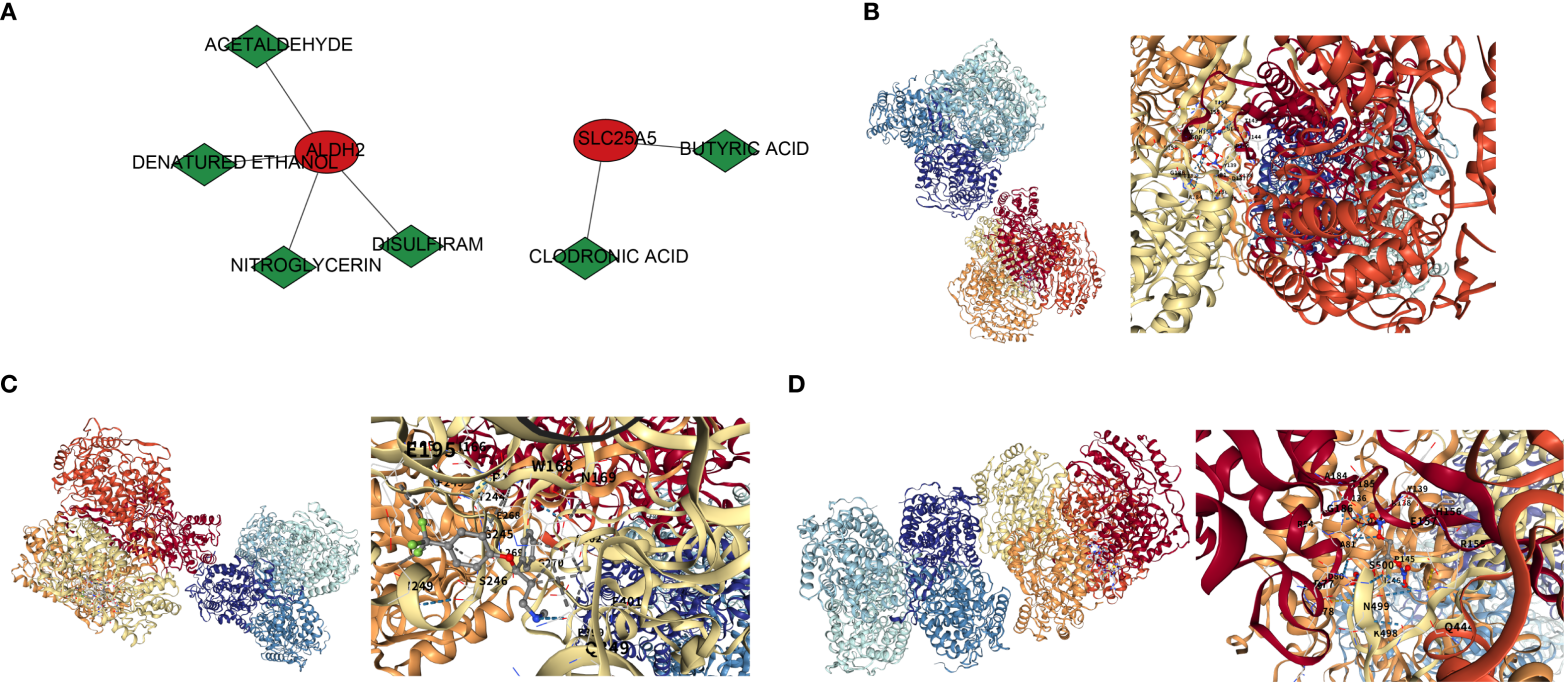
Drug prediction and molecular docking results. **(A)** Drug-gene interaction network. Red circles represent genes, green diamonds represent drug names, and lines connecting drugs and genes indicate regulatory interactions between them. **(B)** Molecular docking of ALDH2 and NITROGLYCERIN. **(C)** Molecular docking of ALDH2 and Selective Serotonin Reuptake Inhibitor. **(D)** Molecular docking of ALDH2 (1CW3) and NITROGLYCERIN.

A total of 27 TFs were predicted in this study. Bioinformatics analysis suggested that STAT1 may target the promoter regions of ALDH2 and SLC25A5, while NKX3–2 may regulate the transcription of IMMT and SLC25A5 (**Supplementary Figure 1A**). Nine target miRNAs were identified by intersecting the predicted miRNAs from the miRWalk and Starbase databases. Based on this, 79 target lncRNAs were predicted, with the following regulatory pairs: HCP5-hsa-miR-27b-3p-SLC25A5, LINC02535-hsa-miR-30b-5p-ALDH2, among others (**Supplementary Figures 1B–D**). Furthermore, drugs corresponding to ALDH2 and SLC25A5 were predicted in the DSigDB database, including four compounds (acetaldehyde, denatured ethanol, nitroglycerin, disulfiram) and two drugs (clodronic acid, butyric acid) (**Figure 6A**). Molecular docking of these drugs with the biomarkers was performed, with ALDH2 (PDB ID: 1nzw) and nitroglycerin showing the most favorable results, exhibiting a docking energy of -6.4 kcal/mol (**Figure 6B**). To validate the docking results, further analysis revealed that the docking energy between ALDH2 and selective serotonin reuptake inhibitors (SSRIs) was 8.2 kcal/mol (**Figure 6C**). When the 3D structure of ALDH2 was replaced with 1CW3, its binding energy with nitroglycerin was 6.3 kcal/mol (**Figure 6D**), which showed minimal change from the original result, further confirming the accuracy of the docking analysis.

### 3.6 Molecular dynamics validation of ALDH2

**Figure 7:**
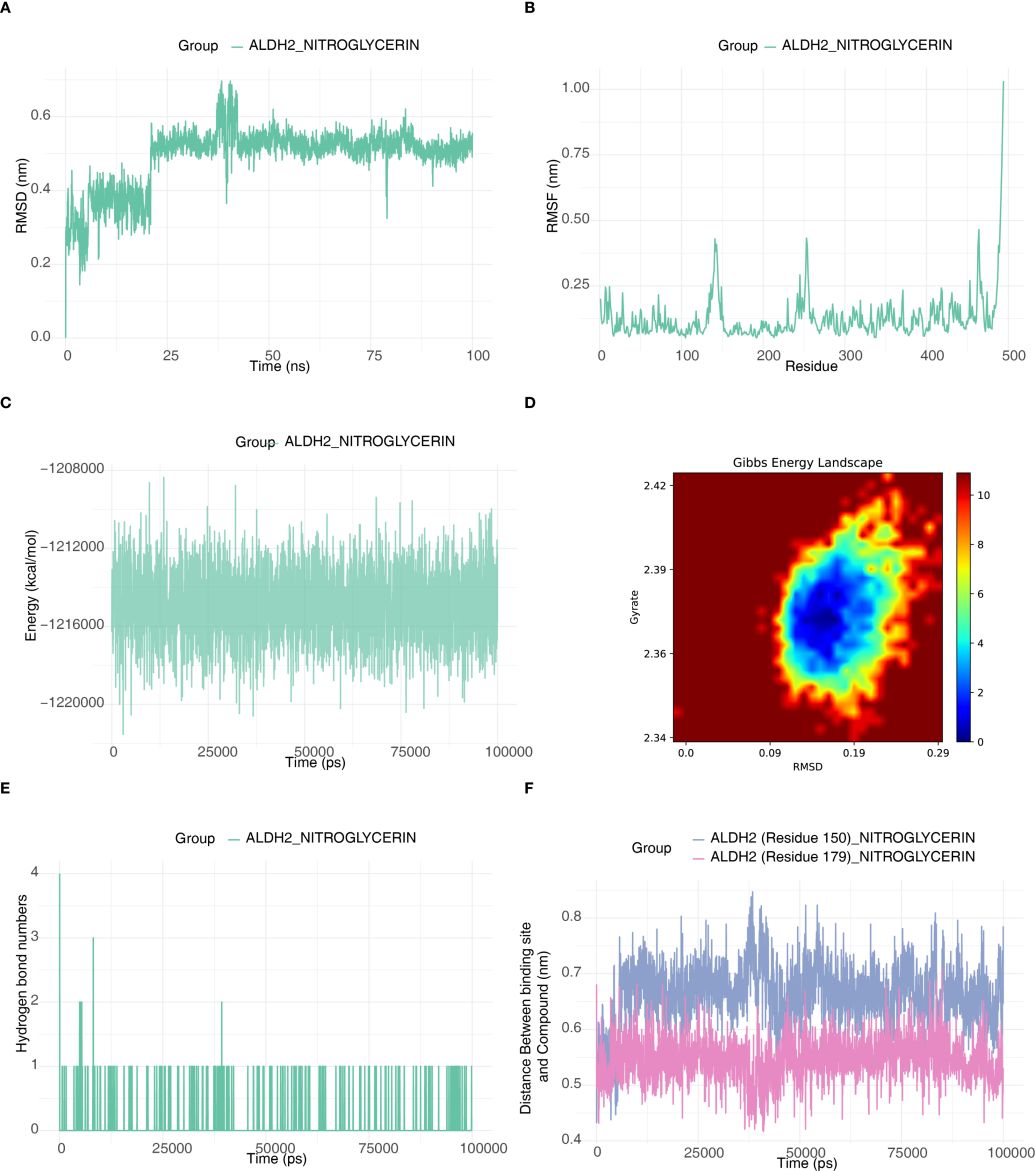
Molecular dynamics validation of ALDH2-NITROGLYCERIN. **(A)** RMSD plot of protein ALDH2. **(B)** RMSF plot of protein ALDH2. **(C)** Energy fluctuation plot between the small molecule drug and the protein. **(D)** Gibbs Free Energy Landscape Diagram of the Interaction between ALDH2 and NITROGLYCERIN. **(E)** Hydrogen bond count plot between the small molecule drug and the protein active site. **(F)** Distance plot between the small molecule drug and the binding site.

This study investigated the conformational changes and energy stability of ALDH2 upon binding to nitroglycerin using 100 ns molecular dynamics simulations. The results showed that the RMSD value of the ALDH2-nitroglycerin system fluctuated between 0.45 and 0.6 nm, indicating that the protein structure reached dynamic equilibrium between 25 and 100 ns and maintained a stable conformation (**Figure 7A**). RMSF analysis revealed that the flexibility of individual residues ranged from 0.05 to 0.4 nm, reflecting local flexibility while ensuring the overall stability of the binding (**Figure 7B**). Energy monitoring demonstrated that the total system energy remained low with minimal fluctuations, and combined with Gibbs free energy landscape analysis, this further confirmed the thermodynamic stability of the complex (**Figures 7C, D**). Hydrogen bond analysis revealed that nitroglycerin formed 1–2 stable hydrogen bonds with the active site of ALDH2, occasionally increasing to 3–4 bonds, highlighting the significance of non-covalent interactions in maintaining binding stability (**Figure 7E**). Additionally, spatial distance monitoring showed that the distances between key binding sites (Residues 150/179) and nitroglycerin stabilized within the ranges of 0.6-0.8 nm and 0.45-0.65 nm, respectively, without a consistent directional change, further confirming the sustained stability of the binding state (**Figure 7F**). In summary, the ALDH2-nitroglycerin complex exhibited stable conformation, favorable thermodynamic properties, and sustained interactions, demonstrating the robustness of their binding.

### 3.7 Biomarkers were localized to different chromosomes and subcellular compartments

**Figure 8:**
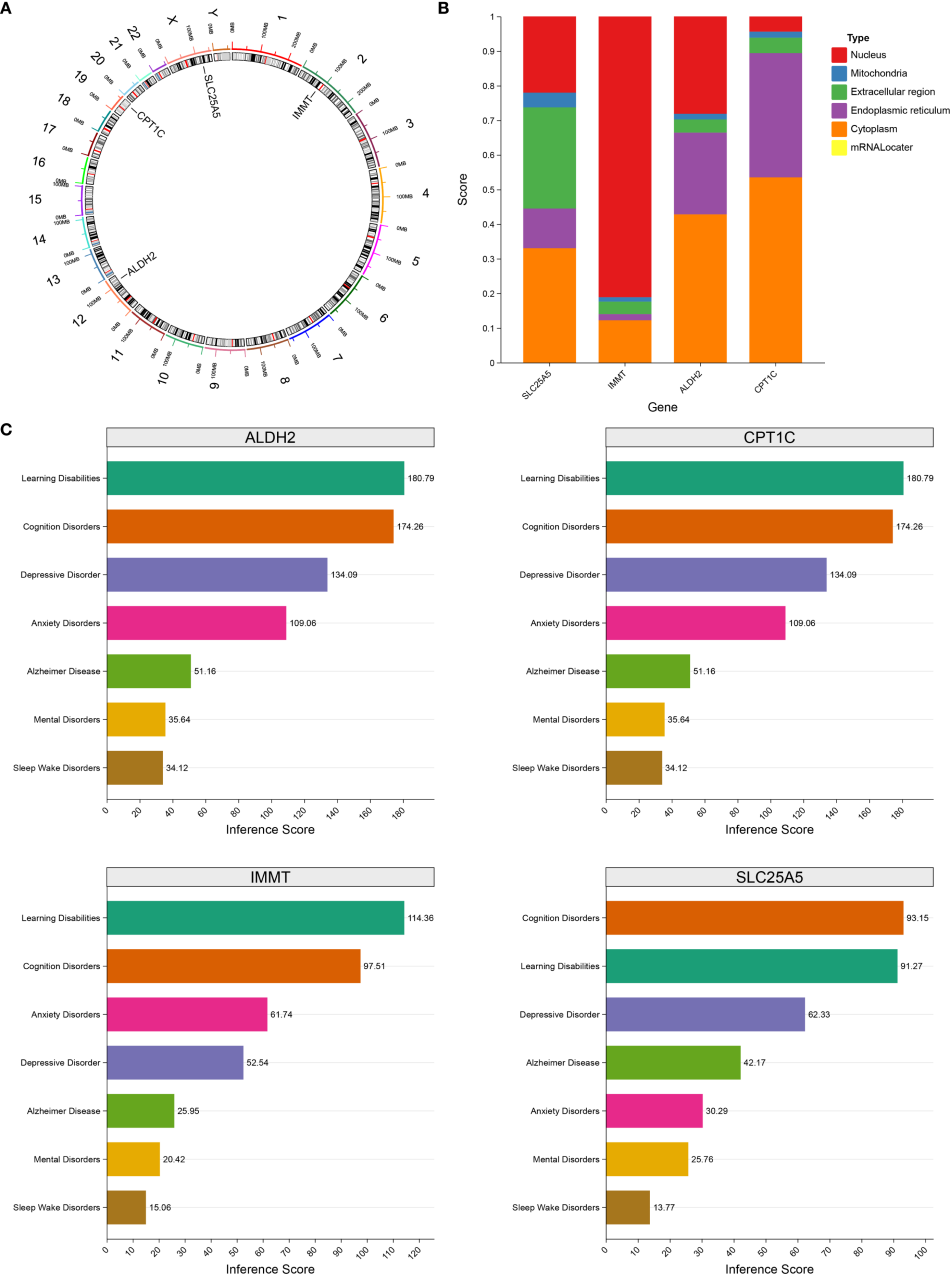
Subcellular localization of biomarkers and chromosome localization analysis. **(A)** The location of biomarkers on chromosomes. **(B)** Subcellular localization scoring of biomarkers. **(C)** The relationship between biomarkers and disease risk. Different colors represented specific diseases respectively: cyan represented Learning Disabilities, orange represented Cognition Disorders, purple represented Depressive Disorder, rose red represented Anxiety Disorders, green represented Alzheimer Disease, yellow represented Mental Disorders, and brown represented Sleep Wake Disorders.

Chromosomal localization analysis indicated that SLC25A5 is located on chromosome X, ALDH2 on chromosome 12, CPT1C on chromosome 19, and IMMT on chromosome 2 (**Figure 8A**). In subcellular localization analysis, IMMT was localized to the nucleus, while SLC25A5, ALDH2, and CPT1C were localized to the cytoplasm (**Figure 8B**). Furthermore, based on the CTD database, the biomarkers displayed higher scores in depression-related diseases, suggesting that they play a pivotal role in depression pathogenesis (**Figure 8C**).

### 3.8 Biomarker expression levels were verified

**Figure 9:**
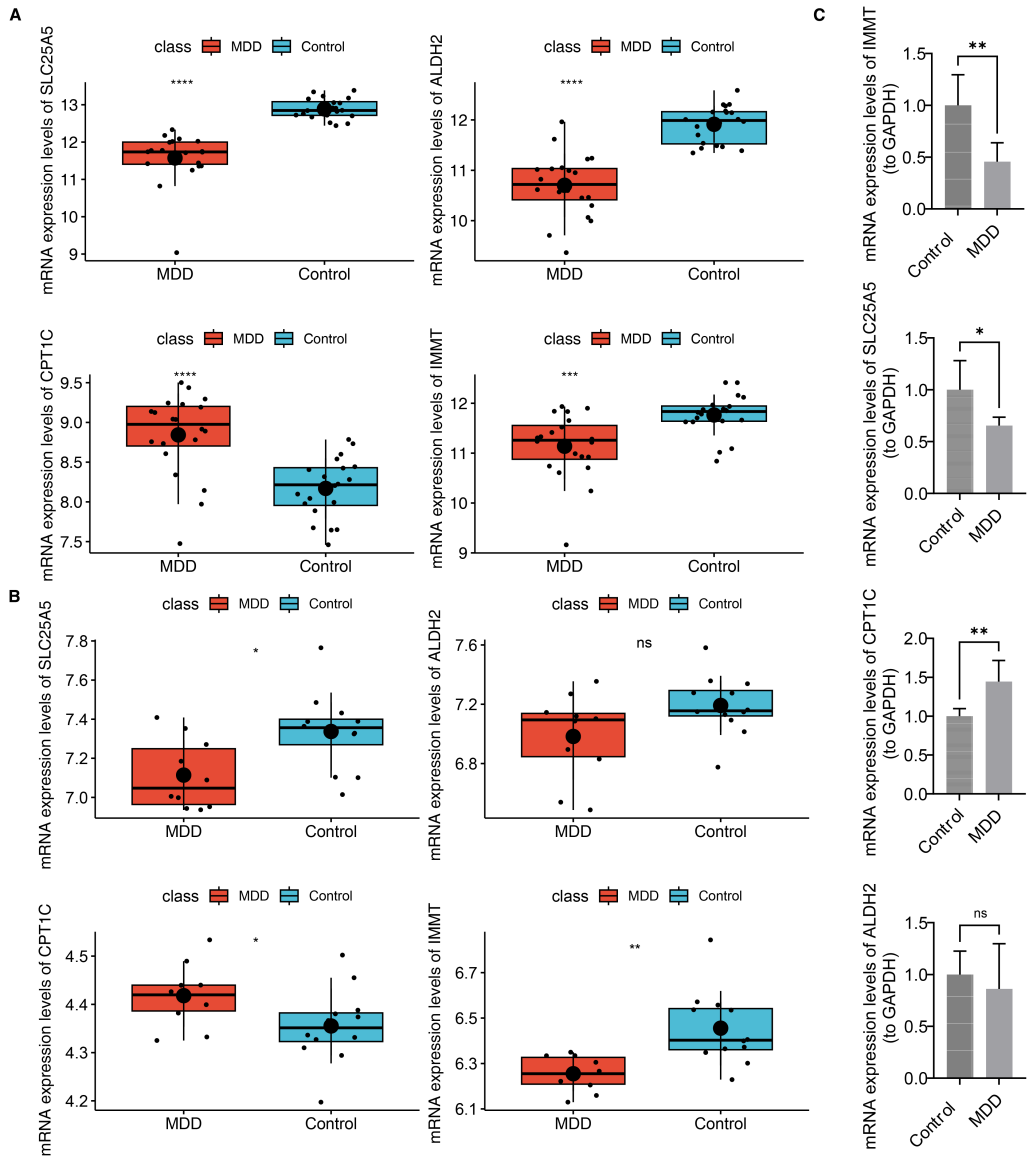
Biomarker expression levels were verified. **(A)** Expression of biomarkers in the training set. ****p-value<0.0001. **(B)** Expression of biomarkers in the validation set. ns represented no significance, *p-value<0.05, **p-value<0.01. **(C)** Validation of biomarker expression in clinical samples by RT-qPCR.

The expression levels of the biomarkers were further validated. In both the training and validation sets, SLC25A5 and IMMT expression were significantly decreased in the MDD group, while CPT1C showed an opposite expression trend. ALDH2 was downregulated in the MDD group in both datasets, although this change was not statistically significant in the validation set (**Figures 9A, B**). RT-qPCR validation revealed that in patients with MDD, IMMT and SLC25A5 expression were significantly lower, while CPT1C expression was markedly elevated compared to controls (p < 0.05) (**Figure 9C**). These findings were consistent with the bioinformatics analysis. Only ALDH2 expression did not show a significant difference (p > 0.05) (**Figure 9C**).

## 4 Discussion

MDD is a debilitating mental disorder characterized by persistent sadness, reduced interest in activities, and various cognitive impairments, which significantly affect the quality of life and functioning of affected individuals. The multifactorial nature of MDD involves genetic, environmental, and neurobiological factors, including changes in brain volume and function, particularly in areas like the hippocampus, which plays a key role in memory and mood regulation (16). Current treatment approaches primarily include psychotherapy and pharmacotherapy, yet a substantial number of patients remain resistant to standard treatments. This underscores the urgent need for novel therapeutic strategies and biomarkers to better understand the complex pathophysiology of MDD (17). Mitochondrial damage and the release of mitochondrial DNA are important markers of age-related inflammation, potentially contributing to the development of depression. Additionally, the age-associated decline in mitochondrial function has been linked to an increased risk of depression (18). Therefore, exploring mitochondrial and aging-related biomarkers may uncover new antidepressant therapies that target the mitochondrial-inflammation axis, offering strategies to reduce the risk of MDD.

This study aims to investigate the role of MRGs and ARGs in MDD using a comprehensive bioinformatics approach. By integrating transcriptomic data from the GEO database, this study identified several candidate biomarkers associated with MDD and conducted functional enrichment analyses to explore their potential roles in the disease’s mechanisms. Among the findings, four key biomarkers—SLC25A5, ALDH2, CPT1C, and IMMT—emerged as significant. These biomarkers could provide valuable insights into the molecular mechanisms underlying MDD, particularly in relation to mitochondrial dysfunction and cellular senescence (19, 20). The results of this study contribute to the existing literature and emphasize the need for further research into the molecular pathways influenced by these biomarkers. Such investigations may pave the way for the development of novel treatment strategies and ultimately improve patient outcomes in MDD (21, 22).

This study highlights the important link between mitochondrial dysfunction and aging in the pathophysiology of MDD. Our findings indicate that the biomarkers SLC25A5, ALDH2, CPT1C, and IMMT exhibit significant expression differences in patients with MDD and are strongly associated with pathways related to mitochondrial dysfunction and aging.

The ANT2 protein, encoded by the SLC25A5 gene, is a key transporter located in the inner mitochondrial membrane, responsible for facilitating the exchange of ADP from the cytosol with ATP from the mitochondrial matrix *via* an “alternate access mechanism” (23). ANT2 plays a key role in the formation and opening of the mitochondrial permeability transition pore (MPTP). Its conformational changes regulate MPTP activity, which, in turn, influences apoptosis. For instance, abnormal activation of ANT2 exacerbates mitochondrial membrane rupture during myocardial ischemia-reperfusion injury (24). In mouse models of anxiety and depression induced by chronic social defeat stress, ANT2 expression in the hippocampus and hypothalamus was significantly upregulated, correlating with mitochondrial dysfunction and the activation of inflammatory pathways (25, 26). In contrast, our PCR results revealed significantly downregulated ANT2 expression in patients with MDD, suggesting that reduced ADP/ATP transport efficiency may impair energy metabolism and compromise neuronal function in brain regions responsible for emotion regulation. The discrepancy between increased ANT2 levels in animal models and decreased levels in patients could stem from differences in disease stages (acute stress versus chronic depression), specific brain regions, or variations in compensatory mechanisms. These findings highlight that ANT2’s role in depression is microenvironment-dependent, and its dysregulated expression could serve as a potential diagnostic biomarker and therapeutic target for MDD through modulation of energy metabolism.

CPT1C (carnitine palmitoyltransferase 1C) is a member of the CPT1 family and is predominantly expressed in the brain, especially in the hypothalamus, hippocampus, and cerebral cortex. It is essential for long-chain fatty acid metabolism, energy homeostasis, lipid regulation, and modulation of neuronal activity (27). CPT1C facilitates the transport of fatty acids into mitochondria for β-oxidation and also regulates neuronal synaptic plasticity, including AMPAR trafficking, through non-catalytic mechanisms, thus influencing neural signal transmission (28). In CPT1C knockout mouse models, researchers observed impaired dendritic spine maturation in the hippocampus, disrupting AMPAR synthesis and trafficking and ultimately compromising spatial learning ability (27). Interestingly, our study found that CPT1C expression was upregulated in patients with MDD. Depression is often associated with synaptic loss in the prefrontal cortex and hippocampus. However, this study observed elevated CPT1C levels, suggesting that this increase may represent a compensatory mechanism to counteract weakened synaptic transmission by enhancing AMPAR synthesis and membrane localization. However, CPT1C’s palmitoyl thioesterase activity must be tightly regulated, as overexpression could disrupt the balance of AMPAR palmitoylation and impair synaptic signaling. CPT1C participates in AMPAR regulation through the BDNF-mTOR pathway, with aberrant BDNF signaling being a core pathological mechanism in depression. The observed upregulation of CPT1C may reflect a compensatory response to reduced BDNF signaling. Nevertheless, under pathological conditions, CPT1C may fail to adequately activate downstream mTOR pathways, preventing functional compensation despite its elevated expression. This contrast underscores the complex and context-dependent roles of CPT1C in synaptic plasticity regulation and the pathology of depression.

ALDH2, a key biomarker identified in this study, plays a pivotal role in managing oxidative stress and detoxifying aldehydes, thereby contributing to neuroprotection. Dysregulation of ALDH2 has been linked to various mood disorders, highlighting its potential relevance in MDD (29). IMMT, involved in mitochondrial dynamics and integrity, has not been extensively studied in the context of MDD, making our findings a novel contribution to understanding its role in depressive pathology (22, 30).

Further GSEA revealed significant involvement of the identified biomarkers in pathways such as ATP-dependent chromatin remodeling and neutrophil extracellular trap formation. These pathways are associated with cellular stress responses and inflammation, both of which are increasingly recognized as key factors in the development of MDD. Notably, ATP-dependent chromatin remodeling is essential for regulating gene expression in response to stress. Disruptions in this process may lead to changes in neuronal plasticity and function, potentially contributing to the mechanisms underlying depression (31, 32). The presence of neutrophil extracellular traps points to an immune-related component in MDD. These structures can influence neuroinflammatory responses and may contribute to the neurodegenerative processes associated with depression (33, 34). These findings suggest that targeting these pathways could offer new therapeutic avenues for treating MDD.

Our drug prediction analysis identified several promising therapeutic agents, with nitroglycerin emerging as a particularly noteworthy candidate due to its favorable molecular docking results with ALDH2. This finding is of particular interest, as nitroglycerin, known for its vasodilatory properties, could potentially influence cerebral blood flow. Additionally, it has been suggested as a possible treatment for certain depressive symptoms, further emphasizing its relevance in this context (35, 36). However, molecular docking alone is insufficient to fully substantiate the therapeutic potential of nitroglycerin in NDD and psychiatric disorders. To bolster the reliability of these findings, further validation through *in vitro* cell experiments and *in vivo* animal models is necessary. Such studies would confirm the interaction between nitroglycerin and its molecular targets, as well as its therapeutic effects from an experimental standpoint. Strengthening the biological relevance of these conclusions through multidimensional experimental data—such as biochemical assays, cellular investigations, and animal behavior analyses—will provide solid scientific support for the potential repurposing of nitroglycerin in treating NDD and psychiatric conditions.

The correlation between the identified biomarkers and immune cell profiles further underscores the critical role of immune dysregulation in the development of MDD. Our immunoinfiltration analysis revealed significantly lower levels of activated B cells, CD8^+^ T cells, and DCs in the peripheral blood of patients with MDD, offering new insights into immune dysfunction in MDD. Notably, this finding mirrors observations in multiple sclerosis (MS), where B cells contribute to neuronal cell death by secreting pro-inflammatory cytokines, such as IL-6 and TNF-α (37). Existing literature supports the association between MDD and abnormal distribution of B cell subsets, characterized by an increase in MHC-II^+^ B cells and a decrease in regulatory B cells (Bregs) and naïve B cells (38), suggesting that B cell dysfunction may represent a common neuroimmune regulatory mechanism in both MDD and MS. Furthermore, a significant positive correlation was found between the expression of the SLC25A5 gene, which encodes mitochondrial adenine nucleotide translocase 2 (ANT2), and the count of activated B cells. This correlation suggests that activated B cells may modulate MPTP opening through ANT2 regulation, potentially disrupting mitochondrial membrane potential and ATP synthesis. Such mitochondrial dysfunction could exacerbate energy metabolism deficits in neural circuits that regulate emotions, thereby intensifying depressive symptoms. These findings highlight the critical interplay between adaptive immunity and mitochondrial bioenergetics in MDD pathogenesis. Our research uncovers novel biomarkers related to mitochondrial function and aging in MDD, emphasizing their potential to guide future treatment strategies and deepen our understanding of the molecular mechanisms underlying this complex disorder. While these results are promising, further validation in larger cohorts and mechanistic studies are required to clarify the significance of these biomarkers in MDD and explore their potential clinical applications.

Despite the preliminary nature of this study, certain limitations remain. First, the sample size validated by RT-qPCR is relatively small, which may restrict the generalizability of the findings. Additionally, only transcriptional validation has been conducted, with no functional validation at the protein level, and the mechanism linking the identified biomarkers to depression remains unexplored. Second, the clinical data of the cohort are incomplete, with missing information such as medication status and body mass index, which may introduce potential confounding factors. Furthermore, the regulatory relationships between TFs, miRNAs, and lncRNAs predicted by bioinformatics tools are hypothetical and require experimental validation.

To address these limitations, future plans involve expanding the sample size and incorporating cohorts with detailed clinical characteristics (such as diagnostic criteria, depression severity, and comorbidities) to enhance the robustness of the results. Protein-level validation will be performed using Western blotting or immunofluorescence, alongside functional studies like gene knockout and animal models to clarify the mechanisms underlying the biomarkers in depression. Additionally, the predicted regulatory relationships will be validated through ChIP assays and overexpression/downregulation functional studies. Longitudinal studies will also be conducted to investigate the dynamic effects of biomarkers on the onset and progression of depressive symptoms. These efforts will provide a stronger theoretical foundation for the diagnosis and intervention of depression.

In conclusion, this study highlights the significant potential of SLC25A5, ALDH2, CPT1C, and IMMT as biomarkers for MDD, elucidating their roles in mitochondrial dysfunction and aging processes. The regulatory networks and immune interactions associated with these biomarkers deepen our understanding of the complex mechanisms underlying MDD. Although certain limitations exist, these findings provide a critical foundation for future studies focused on identifying clinically relevant biomarkers and developing therapeutic targets. Further research into these genes may enhance our understanding of MDD and facilitate the development of more effective treatments for individuals affected by this debilitating condition.

## Data Availability

The original contributions presented in the study are included in the article/**Supplementary Material**. Further inquiries can be directed to the corresponding author.
