# Supplementary material for: Investigating biomarkers of mitochondrial and aging-related genes in major depressive disorder through bioinformatics analysis
Source: Front Psychiatry. 2025 Sep 24;16:1653998. doi: 10.3389/fpsyt.2025.1653998 (PMC12504309; doi:10.3389/fpsyt.2025.1653998)
Supplement: Supplementary file 1 [file Table1.docx]

Supplementary Material

# Supplementary Figures

**Supplementary Figure 1.** Construction of molecular regulatory network. **(A)** TF regulatory network of biomarkers. **(B)** Common miRNAs associated with biomarkers predicted by the mirwalk and starbase databases. **(C)** Common lncRNAs associated with biomarkers predicted by the mirnet and starbase databases. **(D)** mRNA-miRNA-lncRNA regulatory network. Blue diamonds represent lncRNAs, green pentagons represent miRNAs, and red circles represent mRNAs.

# Supplementary Tables

**Supplementary Table 1.** Table of clinical information for GSE201332 dataset.

**Supplementary Table 2.** Table of patients' clinical information for RT-qPCR.

**Supplementary Table 3.** Table of GO and KEGG pathway enrichment results of biomarkers in GSEA enrichment analysis.
