# Supplementary material for: Investigating biomarkers of mitochondrial and aging-related genes in major depressive disorder through bioinformatics analysis
Source: Front Psychiatry. 2025 Sep 24;16:1653998. doi: 10.3389/fpsyt.2025.1653998 (PMC12504309; doi:10.3389/fpsyt.2025.1653998)
Supplement: Supplementary file 5 [file Table5.doc]

Work_dir <- "/YQWLMQ-10108-11"

setwd(Work_dir)

library(limma)

library(edgeR)

library(stringr)

library(ggplot2)

library(tinyarray)

library("tidyverse")

library(idmap1)

library(idmap2)

library(idmap3)

library(ggpubr)

library(survival)

library(survminer)

library(reshape2)

library(ggsci)

library(patchwork)

library("rms")

library(GEOquery)

library("foreign")

library(devtools)

color <- RColorBrewer::brewer.pal(12,'Paired')

color2 <- c("Red3","DodgerBlue4")

main_theme = theme(panel.background=element_blank(), panel.grid=element_blank(),

axis.line.x=element_line(size=.5, colour="black"),

axis.line.y=element_line(size=.5, colour="black"),

axis.ticks=element_line(color="black"),

axis.text.x =element_text(color="black",angle=, size=15),

axis.text.y =element_text(color="black",angle=, size=15),

axis.title.y =element_text(size = 15,colour = "black"),

axis.title.x =element_text(size = 15,colour = "black"),

legend.position="right", legend.background=element_blank(),

legend.key=element_blank(), legend.text= element_text(size=15),

text=element_text(family="sans", size=))

Sys.setenv(LANGUAGE = "en")

options(stringsAsFactors = FALSE)

GEOdownloaddata <- function(GEOnumber,gplnumber){

if(!grepl('^GSE',GEOnumber) | !grepl('^GPL',gplnumber)){ stop('输入的GSE编号或者GPL编号有误') }

gset <- getGEO(GEOnumber, GSEMatrix =TRUE, AnnotGPL=F)

gplloc <- ifelse(any(grepl(gplnumber,names(gset))),grep(gplnumber,names(gset)),1)

gset <- gset[[gplloc]]

if(!is.na(match(gplnumber,unique(idmap1::p2s_df$gpl)))){

ids <- idmap1::getIDs(gplnumber)

}else if(!is.na(match(gplnumber,idmap2::gpl_list$gpl))){

ids <- idmap2::get_soft_IDs(gplnumber)

}else if(!is.na(match(gplnumber,idmap3::gpl_list$gpl))){

ids <- idmap3::get_pipe_IDs(gplnumber)

}else if(!is.null(fData(gset))){

ids <- fData(gset)

if(any(grepl('symbol',tolower(colnames(ids))))){

ids <- ids[,c(1,grep('symbol',tolower(colnames(ids))))]

}else{

print(colnames(ids))

stop('在列名中未找到symbol')

}

}else{

stop('请使用另外的方法下载数据')

}

expr <- exprs(gset)

colnames(ids)[1:2] <- c('ID','symbol')

ids <- ids[match(rownames(expr),ids$ID),]

data <- data.frame(genename=ids[,2],expr)

data$median <- apply(data[,-1],1,median)

data=data[order(data$genename,data$median,decreasing = T),]#

data=data[!duplicated(data$genename),]#

if(length(which(data$genename=='---'| is.na(data$genename)))>0){

data <- data[-which(data$genename=='---'| is.na(data$genename)),]

}

rownames(data)=data$genename#

rnaexpr <- data[,-c(1,ncol(data))]

pdata <- pData(gset)

return(list(rnaexpr,pdata))

}

#############################################################################00_raw_dat###########################################################

dir.create(paste0(Work_dir,"/00_raw_data"))

setwd(paste0(Work_dir,"/00_raw_data"))

options('download.file.method.GEOquery'='libcurl')

GEOnumber="GSE52790"

gplnumber='GPL17976'

gsedata <- GEOdownloaddata(GEOnumber,gplnumber)

rnaCounts <- gsedata[[1]]

pdata <- gsedata[[2]]

save(pdata,rnaCounts,file = 'GSE52790.rda')

MRGs <- read.csv("MitoCarta3.0_gene.csv",header = T)

ARGs <- read.csv("cellSignatures/CellAge_manipulations(866).csv",header = T)

#############################################################################01_DEG_GSE_analysis###########################################################

dir.create(paste0(Work_dir,"/01_DEG_GSE_analysis"))

setwd(paste0(Work_dir,"/01_DEG_GSE_analysis"))

load("D:/xiangmu/1_YQWLMQ-10108-11/00_raw_data/GSE98793.rda")

##GSE19738

load("D:/xiangmu/1_YQWLMQ-10108-11/00_raw_data/GSE19738.rda")

table(pdata$`treatment:ch1`)

pdata <- pdata[pdata$`treatment:ch1` == "none",]

table(pdata$`disease:ch1`)

group_list1 <- ifelse(pdata$`disease:ch1` == "major depressive disorder (MDD)","MDD","Control");table(group_list1)

exper <- rnaCounts[,pdata$geo_accession]

load("D:/xiangmu/1_YQWLMQ-10108-11/00_raw_data/GSE201332_DEG.Rdata")

##GSE201332

group_list1 <- c(rep("MDD",20),rep("Control",20))

group_list1 <- factor(group_list1,levels = c("MDD","Control"))

design <- model.matrix(~0+group_list1)

colnames(design)= levels(group_list1)

rownames(design)= colnames(exper)

max(exper)

fit <- lmFit(exper, design)

constrasts = paste((levels(group_list1)),

collapse = "-");constrasts

cont.matrix <- makeContrasts(contrasts=constrasts,levels = design)

fit2=contrasts.fit(fit,cont.matrix)

fit2=eBayes(fit2)

DEG_TCGA = topTable(fit2, coef=constrasts, n=Inf)

DEG_TCGA = na.omit(DEG_TCGA)

logFC_cutoff <- 0.5

k1 = (DEG_TCGA$adj.P.Val < 0.05)&(DEG_TCGA$logFC < -logFC_cutoff)

k2 = (DEG_TCGA$adj.P.Val < 0.05)&(DEG_TCGA$logFC > logFC_cutoff)

DEG_TCGA$change = ifelse(k1,"DOWN",ifelse(k2,"UP","NOT"))

table(DEG_TCGA$change)

head(DEG_TCGA)

#DEG_TCGA[score_gene,]

write.csv(DEG_TCGA,"DEG_GEO_table.csv")

write.csv(DEG_TCGA[DEG_TCGA$change !="NOT",],"DEG_GEO_table_filler.csv")

table(group1$group)

library(ggplot2)

library(tinyarray)

group1 <- data.frame(id = colnames(exper),group = group_list1) ; group1 <- group1[order(group1$group),]

exper <- exper[,match(group1$id,colnames(exper))]

identical(colnames(exper),group1$id)

DEG_mrna = rownames(DEG_TCGA)[DEG_TCGA$change !="NOT"]

#

h3 = draw_heatmap(exper[DEG_mrna,],group1$group,n_cutoff = 2,legend = T,annotation_legend = T,cluster_cols = F,

scale = T,color_an = c( "#FC8D62","#66C2A5","red"),color = (grDevices::colorRampPalette(c("#377EB8", "white", "#E41A1C")))(100))

h3

# ggsave("fig.differential_gene_heat_map.pdf", h3, width = 10, height = 6)

library(ComplexHeatmap)

con_gen <- as.data.frame(t(exper[DEG_mrna,]))

gene <- as.data.frame(t(apply(con_gen, 2, scale)))

colnames(gene) <- row.names(con_gen)

col_fun = circlize::colorRamp2(c(-2, 0, 2), c("#377EB8", "white", "#E41A1C"))

ha = HeatmapAnnotation(

group = group1$group)

#set.seed(14)

pdf("fig.differential_gene_heat_map.pdf", width = 10, height = 8)

densityHeatmap(gene)%v%

Heatmap(gene,col = col_fun,

cluster_rows = T,

cluster_columns = F,

show_column_names = F,

show_row_names = F, top_annotation = ha)

dev.off()

library(ggVolcano)

a <- as.numeric(table(DEG_TCGA$change)[c(1,3)])

DEG_TCGA <- add_regulate(DEG_TCGA, log2FC_name = "logFC",

fdr_name = "adj.P.Val",log2FC = logFC_cutoff, fdr = 0.05)

table(duplicated(rownames(DEG_TCGA)))

DEG_TCGA$gene <- rownames(DEG_TCGA)

colnames(DEG_TCGA)

pdf("fig.differential_gene_volcano_map.pdf", width = 10, height = 8)

gradual_volcano(DEG_TCGA, x = "log2FoldChange", y = "padj",

label = "gene", label_number = 10, output = FALSE,log2FC_cut = 0.5) + ggtitle(paste0(" Down",a[1]," Up",a[2]))

dev.off()

save(exper,DEG_TCGA,group1,DEG_mrna,file = "GSE201332_DEG.Rdata")

#######################################02_con_gene_analysis######################################################

dir.create(paste0(Work_dir,"/02_con_gene_analysis"))

setwd(paste0(Work_dir,"/02_con_gene_analysis"))

load("D:/xiangmu/1_YQWLMQ-10108-11/01_DEG_GSE_analysis/GSE201332_DEG.Rdata")

table(DEG_TCGA$change)

library("ggvenn")

genes <- list(DEG_mrna=DEG_mrna,

ARGs=ARGs$gene,

MRGs=MRGs$Symbol)

plot <- ggvenn(genes,show_percentage = T,

stroke_color = "white",

stroke_size = 0.5,

#fill_color = c("#E41A1C","#1E90FF"),

#set_name_color =c("#E41A1C","#1E90FF","#FF8C00"),

set_name_size = 6,text_size=5)

plot

ggsave("fig.DEG_con_gene_filler.pdf", plot, width = 8, height = 8)

con_gene <- intersect(DEG_mrna,intersect(ARGs$gene,MRGs$Symbol))

load("D:/xiangmu/1_YQWLMQ-10108-11/00_raw_data/GSE52790_DEG.Rdata")

DEG_TCGA[con_gene,]

write.csv(con_gene,file = "DEG_con_gene.csv")

library(clusterProfiler)

library(ggplot2)

library(enrichplot)

library(org.Mm.eg.db)

library(enrichplot)

library(GOplot)

R.utils::setOption( "clusterProfiler.download.method",'auto' )

gene<-bitr(con_gene,fromType = 'SYMBOL',toType = 'ENTREZID',OrgDb = 'org.Hs.eg.db')

GO<-enrichGO(

gene$ENTREZID,

OrgDb = 'org.Hs.eg.db',

keyType = "ENTREZID",

ont = "ALL",

pvalueCutoff = 0.5,

pAdjustMethod = "BH",

qvalueCutoff = 0.5,

readable = TRUE

)

KEGG<-enrichKEGG(

gene$ENTREZID,

organism = "hsa",

keyType = "kegg",

pvalueCutoff = 0.5,

pAdjustMethod = "BH",

qvalueCutoff = 0.5,

)

barplot(GO, split="ONTOLOGY")+facet_grid(ONTOLOGY~., scale="free")

go <- as.data.frame(GO)

write.csv(file = "DEG_con_GO_enrich.csv",go[go$pvalue < 0.05 & go$Count > 1,])

boxdata <- go[go$pvalue < 0.05 & go$Count > 1,]

colnames(boxdata)

boxdata$LogP <- log(boxdata$pvalue)

boxdata$Count <- sapply(boxdata$Count,function(x){as.numeric(x)})

#boxdata <- boxdata[order(boxdata$Count,decreasing = T),]

boxdata <- rbind(subset(boxdata,ONTOLOGY=='BP')[1:12,],

subset(boxdata,ONTOLOGY=='CC')[1:10,],

subset(boxdata,ONTOLOGY=='MF')[1:10,]) %>% na.omit(x)

boxdata <- boxdata[order(boxdata$Count,decreasing = F),]

boxdata$Description <- factor(boxdata$Description,levels = boxdata$Description)

library(treemap)

pdf('Fig.DEG_con_gene_black.enrichGO.pdf',width = 12,height =8)

treemap(boxdata,

index="Description",

vSize="Count",

vColor="pvalue",

type="value",

palette='-Blues',

#palette = "Set2",

fontsize.labels=c(10, 10),

align.labels=list(c("center", "center"), c("left", "top")),

border.col="black",

border.lwds=c(2,2)

)

dev.off()

#GO_circ

boxdata <- boxdata[order(boxdata$Count,decreasing = T),]

GOplotIn<-boxdata[1:10,c(2,3,7,9)]

GOplotIn$geneID <-str_replace_all(GOplotIn$geneID,'/',',')

names(GOplotIn)<-c('ID','Term','adj_pval','Genes')

GOplotIn$Category = "BP"

gene_fc <- DEG_TCGA[con_gene,] %>% na.omit(.)

genedata<-data.frame(ID=row.names(gene_fc),logFC=gene_fc$logFC)

circ<-GOplot::circle_dat(GOplotIn,genedata)

chord<-chord_dat(data = circ,genes = genedata)

pdf("fig.DEG_con_gene_GO_circ.pdf",height = 14,width = 14)

GOChord(

data = chord,

space = 0.02,

limit = c(1,1),

gene.order = 'logFC',

gene.space = 0.25,

gene.size = 3,

ribbon.col = RColorBrewer::brewer.pal(n = 10, name = "Paired")[1:10],

#ribbon.col = brewer.pal(length(GOplotIn$Term)),#GO Term colors

process.label = 8 #GO

)+#ribbon.col = mycol[1:n])+

ggtitle("GO Pathway") +

theme(plot.title = element_text(size = 30,hjust = 0.5))

dev.off()

barplot(KEGG,title = 'KEGG Pathway')

kegg <- as.data.frame(KEGG)

write.csv(file = "DEG_con_kegg.csv",kegg[kegg$pvalue < 0.05 & kegg$Count > 1,])

kegg <- kegg[kegg$pvalue < 0.05 & kegg$Count > 1,]

dim(kegg)[1]

boxdata <- kegg

boxdata$LogP <- log(boxdata$pvalue)

boxdata$Count <- sapply(boxdata$Count,function(x){as.numeric(x)})

boxdata <- boxdata[order(boxdata$Count,decreasing = F),]

boxdata$Description <- factor(boxdata$Description,levels = boxdata$Description)

pdf('Fig.DEG_con.black.enrichKEGG.pdf',width = 10,height =6)

treemap(boxdata,

index="Description",

vSize="Count",

vColor="pvalue",

type="value", #

palette='-Blues',

#palette = "Set2",

fontsize.labels=c(10, 10), #

align.labels=list(c("center", "center"), c("left", "top")), #

border.col="black", #

border.lwds=c(2,2)#

)

dev.off()

################kegg_circ

gene_fc <- DEG_TCGA[con_gene,] %>% na.omit(.)

genesig1<-data.frame(ID=row.names(gene_fc),logFC=gene_fc$log2FoldChange)

genesig1 <- genesig1[genesig1$ID %in% gene$SYMBOL,]

genesig1 <- genesig1[match(gene$SYMBOL,genesig1$ID),]

identical(gene$SYMBOL,genesig1$ID)

genesig1$ID <- gene$ENTREZID

genesig1 <- na.omit(genesig1)

genesig1 <- genesig1[,c('ID','logFC')]

rownames(genesig1) <- 1:nrow(genesig1)

egokegg <- kegg[1:10,]

#egobp[1,]

kegg1 <- data.frame(Category='KEGG',

ID = egokegg$ID,

Term = egokegg$Description,

Genes = gsub("/", ", ", egokegg$geneID),

adj_pval = egokegg$p.adjust)

circkegg <- circle_dat(kegg1,genesig1)

n =10

mycol <- brewer.pal(10, "Set3")

mycol <- mycol[c(2,4,5)]

#mycol <- brewer.pal(2, "Set2")

chor <- chord_dat(circkegg, genesig1,kegg1$Term)

row.names(chor) <- bitr(row.names(chor),fromType = 'ENTREZID',toType = 'SYMBOL',OrgDb = 'org.Hs.eg.db') %>% .$SYMBOL

#head(chord)

pdf('Fig.DEG_con_enrichKEGG_circ.pdf',width = 13,height = 13)

GOChord(chor,

space = 0.02, #

gene.order = 'logFC',

lfc.col = c('darkgoldenrod1', 'black', 'cyan1'), #

gene.space = 0.3, #

gene.size = 3, #

border.size = 0.1, #

process.label = 8,

ribbon.col = RColorBrewer::brewer.pal(n = 10, name = "Paired")[1:10]

)+#ribbon.col = mycol[1:n])+ #

ggtitle("KEGG Pathway") + #

theme(plot.title = element_text(size = 30,hjust = 0.5)) #

dev.off()

################05_Machine_learning####################################################

dir.create(paste0(Work_dir,"/04_Machine_learning"))

setwd(paste0(Work_dir,"/04_Machine_learning"))

library(glmnet)

load("D:/xiangmu/1_YQWLMQ-10108-11/01_DEG_GSE_analysis/GSE201332_DEG.Rdata")

DEG_TCGA[con_gene,]

identical(colnames(exper),group1$id)

x=as.matrix(t(exper[con_gene,]))

y=ifelse(group1$group == "MDD",1,0)

set.seed(30)

fit <- glmnet(x, y, family = "binomial", maxit = 5000)

cvfit <- cv.glmnet(x, y, family="binomial", maxit = 5000)

pdf(file = "Fig.lasso_lambda.pdf")

plot(fit, xvar = "lambda", label = TRUE)

dev.off()

pdf(file = "Fig.lasso_lcvfit.pdf")

plot(cvfit)

abline(v=log(c(cvfit$lambda.min,cvfit$lambda.1se)),lty="dashed")#8.8是文字在横轴的位置，可以AI调整

text(log(cvfit$lambda.min),mean(cvfit[["cvm"]]),cex=1.5,

labels = paste0('lambda.min = \n',round(cvfit$lambda.min,4)))#8.8是文字在横轴的位置，可以AI调整

text(log(cvfit$lambda.1se),mean(cvfit[["cvm"]]),cex=1.5,

labels = paste0('lambda.lse = \n',round(cvfit$lambda.1se,4)))

dev.off()

coef <- coef(fit, s = cvfit$lambda.min)

index <- which(as.matrix(coef)!= 0)

actCoef <- coef[index]

lassoGene=row.names(coef)[index];lassoGene

if (length(lassoGene)<=1){

next

}

myCoefs <- coef(cvfit, s="lambda.min")

#coef <- read.table("lasso_coef.txt",sep = "\t",header = T)

write.table(as.matrix(myCoefs),"lasso_coef.txt",sep = "\t")

#roc

library(RColorBrewer)

mycol <- brewer.pal(n = 10, name = "Paired")

mycol <- c("slateblue","seagreen3","dodgerblue","firebrick1","lightgoldenrod","magenta","orange2")

library("pROC")

myoverlap <- lassoGene[-1]

df <- input[,c("group",myoverlap)]

df <- input[,c("group",myoverlap)]

for (i in colnames(df)[-1]) {

pdf(paste(i,"_GSE201332_ROC.pdf",sep = "-"),height=6,width=6)

x <- plot.roc(df[,1],df[,i],ylim=c(0,1),xlim=c(1,0),

smooth=F,

ci=TRUE,

main= i,

#print.thres="best",

col="red",#

lwd=2, #

legacy.axes=T,

print.auc=TRUE)#

dev.off()

}

auc.out <- c()

pdf("Fig.ALL_train_ROC.pdf",height=6,width=6)

x <- plot.roc(df[,1],df[,2],ylim=c(0,1),xlim=c(1,0),

smooth=F,

ci=T,

main="",

#print.thres="best",

col=mycol[2],#

lwd=2, #

legacy.axes=T)#

ci.lower <- round(as.numeric(x$ci[1]),1) #

ci.upper <- round(as.numeric(x$ci[3]),1) #

auc.ci <- c(colnames(df)[2],round(as.numeric(x$auc),1),paste(ci.lower,ci.upper,sep="-"))

auc.out <- rbind(auc.out,auc.ci)

for (i in 3:ncol(df)){

x <- plot.roc(df[,1],df[,i],

add=T, #

smooth=F,

ci=T,

col=mycol[i],

lwd=2,

legacy.axes=T)

ci.lower <- round(as.numeric(x$ci[1]),3)

ci.upper <- round(as.numeric(x$ci[3]),3)

auc.ci <- c(colnames(df)[i],round(as.numeric(x$auc),2),paste(ci.lower,ci.upper,sep="-"))

auc.out <- rbind(auc.out,auc.ci)

}

auc.out <- as.data.frame(auc.out)

colnames(auc.out) <- c("Name","AUC","AUC CI")

legend.name <- paste(colnames(df)[2:length(df)],"AUC",auc.out$AUC,sep=" ")

legend("bottomright",

legend=legend.name,

col = mycol[2:length(df)],

lwd = 2,

bty="n")

dev.off()

auc.out<- auc.out

write.table(auc.out,"auc.out_train.txt",sep="\t",quote = F,row.names = F,col.names = T)

#

exp <- (exper[myoverlap,])

exp <- as.data.frame(t(exp))

exp$class <- factor(group1$group)

pl <- list()

for (i in myoverlap) {

dat <- exp[,c("class",i)]

pl[[i]] <- ggboxplot(dat, x = "class", y = i, fill = "class", size = 0.5,palette = "npg",notch = F,

add = c("jitter","mean_sd"),add.params=list(size=1)) +

labs(y = paste0(i," gene expression"), x = "")+

rotate_x_text(angle =0 )+

stat_compare_means(aes(group = class),label = "p.signif",method = "anova")

# pdf(paste0(i,'_gene_expression_box(Train).pdf'),width=6,height = 6)

# print(pl[[i]],newpage = FALSE)

# dev.off()

}

p = ggarrange(pl[[1]],pl[[2]],pl[[3]],pl[[4]],ncol = 2, nrow = 2)

p

ggsave("fig.all_gene_combine_box(Train).pdf",p, width = 8, height = 6)

##

load("D:/xiangmu/1_YQWLMQ-10108-11/00_raw_data/GSE52790_DEG.Rdata")

DEG_TCGA[myoverlap,]

x=as.matrix(t(exper[myoverlap,]))

y=ifelse(group1$group == "MDD",1,0)

input <- as.data.frame(x)

input$group <- factor(y)

input <- input[,c(ncol(input),1:ncol(input)-1)]

df <- input[,c("group",myoverlap)]

for (i in colnames(df)[-1]) {

pdf(paste(i,"_GSE52790_ROC.pdf",sep = "-"),height=6,width=6)

x <- plot.roc(df[,1],df[,i],ylim=c(0,1),xlim=c(1,0),

smooth=F, #

ci=TRUE,

main= i,

#print.thres="best", #

col="red",#

lwd=2, #

legacy.axes=T,

print.auc=TRUE)#

dev.off()

}

auc.out <- c()

pdf("test_ROC_gene.pdf",height=6,width=6)

x <- plot.roc(df[,1],df[,2],ylim=c(0,1),xlim=c(1,0),

smooth=F, #

ci=T,

main="",

#print.thres="best", #

col=mycol[2],

lwd=2, #

legacy.axes=T)

ci.lower <- round(as.numeric(x$ci[1]),1) #

ci.upper <- round(as.numeric(x$ci[3]),1) #

auc.ci <- c(colnames(df)[2],round(as.numeric(x$auc),1),paste(ci.lower,ci.upper,sep="-"))

auc.out <- rbind(auc.out,auc.ci)

for (i in 3:ncol(df)){

x <- plot.roc(df[,1],df[,i],

add=T, #

smooth=F,

ci=T,

col=mycol[i],

lwd=2,

legacy.axes=T)

ci.lower <- round(as.numeric(x$ci[1]),3)

ci.upper <- round(as.numeric(x$ci[3]),3)

auc.ci <- c(colnames(df)[i],round(as.numeric(x$auc),2),paste(ci.lower,ci.upper,sep="-"))

auc.out <- rbind(auc.out,auc.ci)

}

auc.out <- as.data.frame(auc.out)

colnames(auc.out) <- c("Name","AUC","AUC CI")

#write.table(auc.out,"auc_output.xls",sep="\t",quote = F,row.names = F,col.names = T)

legend.name <- paste(colnames(df)[2:length(df)],"AUC",auc.out$AUC,sep=" ")

legend("bottomright",

legend=legend.name,

col = mycol[2:length(df)],

lwd = 2,

bty="n")

dev.off()

exp <- (exper[myoverlap,])

exp <- as.data.frame(t(exp))

exp$class <- factor(group1$group)

pl <- list()

for (i in myoverlap) {

dat <- exp[,c("class",i)]

pl[[i]] <- ggboxplot(dat, x = "class", y = i, fill = "class", size = 0.5,palette = "npg",notch = F,

add = c("jitter","mean_sd"),add.params=list(size=1)) +

labs(y = paste0(i," gene expression"), x = "")+

rotate_x_text(angle =0 )+

stat_compare_means(aes(group = class),label = "p.signif",method = "t.test")

# pdf(paste0(i,'_gene_expression_box(Train).pdf'),width=6,height = 6)

# print(pl[[i]],newpage = FALSE)

# dev.off()

}

p = ggarrange(pl[[1]],pl[[2]],pl[[3]],pl[[4]],ncol = 2, nrow = 2)

p

ggsave("fig.all_gene_combine_box(Test).pdf",p, width = 8, height = 6)

#####################################################################06_ANN_analysis###########################

dir.create(paste0(Work_dir,"/05_ANN_analysis"))

setwd(paste0(Work_dir,"/05_ANN_analysis"))

library(neuralnet)

library(NeuralNetTools)

load("GSE201332_DEG.Rdata")

#load("GGSE26168_DEG.Rdata")

neuralnet(formula, data, hidden = 1, threshold = 0.01,stepmax = 1e+05,

rep = 1, startweights = NULL, err.fct = "sse",linear.output = TRUE,

learningrate=NULL,algorithm = "rprop+")

x=as.matrix(t(exper[myoverlap,]))

y=ifelse(group1$group == "MDD",1,0)

input <- as.data.frame(x)

input$group <- factor(y)

input <- input[,c(ncol(input),1:ncol(input)-1)]

df <- input

nn_model <- neuralnet(group~SLC25A5+ALDH2+CPT1C+IMMT,data=df,hidden=4,err.fct = 'ce',linear.output = F)

nn_model$result.matrix

write.csv(nn_model$result.matrix,file = "NN_result.matrix.csv")

head(nn_model$generalized.weights[[1]])

pdf("neural_network_plot1.pdf")

print(plot(nn_model))

dev.off()

pdf("neural_network_plot.pdf")

par(cex = 0.6)

plotnet(nn_model,pos_col = "red", neg_col = "grey")

dev.off()

#

predicted_nn <- compute(nn_model, df)$net.result

predicted_labels_nn <- ifelse(predicted_nn > 0.5, 1, 0)

predict_data <- data.frame(true_labels = df$group,predicted_labels=predicted_labels_nn[,2])

roc <- roc(predict_data$true_labels,as.numeric(predict_data$predicted_labels))

pdf("train_ROC_ANN.pdf",height=6,width=6)

plot(roc, print.auc=TRUE, auc.polygon=TRUE,

grid=c(0.1, 0.1),grid.col=c("green", "red"),

max.auc.polygon=TRUE,auc.polygon.col="skyblue",

print.thres=TRUE,main='Train-ROC-curve')

dev.off()

#

load("GSE52790_DEG.Rdata")

exp <- exper[myoverlap,]

exp <- as.data.frame(t(exp))

exp$group <- ifelse(group1$group == "MDD",1,0)

df <- exp[,c(ncol(exp),1:ncol(exp)-1)]

predicted_nn <- compute(nn_model, df)$net.result

predicted_labels_nn <- ifelse(predicted_nn > 0.5, 1, 0)

predict_data <- data.frame(true_labels = df$group,predicted_labels=predicted_labels_nn[,2])

roc <- roc(predict_data$true_labels,as.numeric(predict_data$predicted_labels))

roc <- roc(df$group,as.numeric(df$all))

pdf("test_ROC_ANN.pdf",height=6,width=6)

plot(roc, print.auc=TRUE, auc.polygon=TRUE,

grid=c(0.1, 0.1),grid.col=c("green", "red"),

max.auc.polygon=TRUE,auc.polygon.col="skyblue",

print.thres=TRUE,main='Test-ROC-curve')

dev.off()

#################################06_Single_gene_enrichment_analysis###################################################

dir.create(paste0(Work_dir,"/06_Single_gene_enrichment_analysis"))

setwd(paste0(Work_dir,"/06_Single_gene_enrichment_analysis"))

load("GSE201332_DEG.Rdata")

library(ReactomePA)

library(clusterProfiler)

library(ggplot2)

library(enrichplot)

library(RColorBrewer)

library(EnhancedVolcano)

library(org.Hs.eg.db)

library(GOplot)

library(ggplot2)

source("gesa2.r")

R.utils::setOption( "clusterProfiler.download.method",'auto' )

as <- myoverlap

LUAD_expr <- as.data.frame(exper)

i <- "SLC25A5"

for (i in as) {

target.exps <- LUAD_expr[i,]

other.expr <- LUAD_expr[-which(rownames(LUAD_expr)==i),]

# pearson

sgcor <- as.data.frame(cor(t(other.expr), t(target.exps))) #

colnames(sgcor) <- "r_pearson"

sgcor$pval_pearson <- apply(other.expr, 1, function(x) (cor.test(x, t(target.exps))$p.value))

cors <- sgcor

cors$SYMBOL <- rownames(other.expr)

gene = bitr(cors$SYMBOL ,fromType = 'SYMBOL',toType = 'ENTREZID',OrgDb = 'org.Hs.eg.db')

cors <- cors %>%

inner_join(gene,by = "SYMBOL")

cors <- cors %>%

arrange(desc(r_pearson))%>% na.omit(.)

head(cors)

gene_list <- cors$r_pearson

names(gene_list) <- cors$ENTREZID

head(gene_list)

gene_list <- gene_list[!duplicated(gene_list)]

gse.GO <- gseGO(geneList = gene_list,

ont = "ALL",

keyType = "ENTREZID",

OrgDb = org.Hs.eg.db,

pAdjustMethod = "BH",

pvalueCutoff = 0.5)

gse.GO1 <- as.data.frame(gse.GO)

gse.GO1 <- gse.GO[gse.GO$pvalue < 0.05,]

write.csv(file = paste0(i,"_go_gesa.csv"),gse.GO[gse.GO$pvalue < 0.05,])

#team_id <- grep("immune| T cell | B cell",gse.GO$Description)

p <- gseaplot3(gse.GO,1:10,pvalue_table = F,rel_heights = c(1.5, 0.5, 0.5),color= brewer.pal(10,'Paired'))

pdf(paste0(i,"_go_gesa.pdf"), width = 10, height = 10)

print(p)

dev.off()

gse.KEGG <- gseKEGG(geneList = gene_list,

organism = "hsa",

pvalueCutoff = 0.8)

gse.kegg <- as.data.frame(gse.KEGG)

write.csv(file = paste0(i,"_kegg_gesa.csv"),gse.kegg[gse.kegg$pvalue < 0.05,])

p2 <- gseaplot3(gse.KEGG,1:10, pvalue_table = F,rel_heights = c(1.5, 0.5, 0.5),color= brewer.pal(10,'Paired'))

pdf(paste0(i,"_kegg_gesa.pdf"), width = 10, height = 10)

print(p2)

dev.off()

}

###########################################06_score_gene_local_analysis###############################################

dir.create(paste0(Work_dir,"/07_score_gene_local_analysis"))

setwd(paste0(Work_dir,"/07_score_gene_local_analysis"))

#chr local

library(RCircos)

data("UCSC.HG38.Human.CytoBandIdeogram")

head(UCSC.HG38.Human.CytoBandIdeogram)

#######mrna

RCircos.Set.Core.Components(UCSC.HG38.Human.CytoBandIdeogram,

chr.exclude <- NULL,

tracks.inside = 10,

tracks.outside = 0);

RCircos.List.Plot.Parameters()

mrna_chr <- read.csv("score_gene_information_result.csv",header = T,check.names = F)

mrna_chr$CHR <- paste("chr",mrna_chr$CHR,sep ="")

gene <- data.frame(chromosome=mrna_chr$CHR,

start=mrna_chr$Start.Site,

stop=mrna_chr$End.Site,

gene=mrna_chr$gen )

table(gene$chromosome)

pdf("fig.score_gene_chr_local_analysis.pdf", height=6, width=6, compress=TRUE);

RCircos.Set.Plot.Area();

RCircos.Chromosome.Ideogram.Plot(tick.interval=50);

RCircos.Ideogram.Tick.Plot(tick.interval=50)

RCircos.Label.Chromosome.Names(chr.name.pos=NULL)

RCircos.Draw.Chromosome.Ideogram(ideo.pos=NULL, ideo.width=NULL)

RCircos.Highligh.Chromosome.Ideogram(highlight.pos=NULL, highlight.width=NULL)

side <- "in";

track.num <- 1;

RCircos.Gene.Connector.Plot(gene, track.num, side);

name.col <- 4;

track.num <- 2;

RCircos.Gene.Name.Plot(gene, name.col,track.num, side);

dev.off()

#############################08_Imm_cell_analysis######################################################

dir.create(paste0(Work_dir,"/08_Imm_cell_analysis"))

setwd(paste0(Work_dir,"/08_Imm_cell_analysis"))

library("GSVA")

load("28Immune_Cell.Rdata")

ssgsva <- gsva(as.matrix(exper),list, method = "ssgsea")

ssgsva <- ssgsva[,match(group1$id,colnames(ssgsva))]

group_list <- as.factor(group1$group)

identical(group1$id,colnames(ssgsva))

write.table(ssgsva,file = "TCGA_Immune_infiltration.txt",quote = F,sep = "\t")

ssgsva <- read.table("TCGA_Immune_infiltration.txt",header = T,sep = '\t',check.names = F)

Ssgsva <- data.frame(t(ssgsva))

colnames(Ssgsva) <- gsub('\\.',' ',colnames(Ssgsva))

row.names(Ssgsva) <- gsub('\\.','-',row.names(Ssgsva))

colnames(Ssgsva)[9:10] <-c("Effector memory CD4 T cell","Effector memory CD8 T cell")

rownames(Ssgsva)

data2 <- Ssgsva %>%

add_column(class = group1$group, .before = 1) %>%

gather("cell_types","percentages", -class)%>%

mutate(cell_types = factor(cell_types, levels = colnames(Ssgsva))) %>%

mutate(class = factor(class, levels = c("MDD","Control")))

data3 <- data2 %>%

group_by(class,cell_types) %>%

summarise("Mx" =max(percentages)) %>%

group_by(cell_types) %>%

summarise(lable = max(Mx))

x = c()

xend = c()

for(i in 1:length(unique(data2$cell_types))){

x[i] = i - 0.2

xend[i] = i + 0.2}

y = data3$lable +0.01

yend = data3$lable +0.01

line <- data.frame(x, xend, y, yend)

library(ggthemes)

P <- ggplot()+

geom_violin(data = data2, aes(x = cell_types, y = percentages,fill = class),

position = position_dodge(1),scale = "width")+

geom_boxplot(data = data2, aes(cell_types, percentages,fill = class),

position = position_dodge(1),width=.4, outlier.shape = NA)+

stat_summary(data = data2, aes(cell_types, percentages,fill = class),

fun = "median",geom = "point",shape = 16, size = 2,

color = "white",position = position_dodge(1))+

ggsci::scale_fill_aaas()+

ggpubr::stat_compare_means(data = data2,aes(cell_types, percentages,group = class,

),label = "p.signif",

method = "wilcox.test",label.y = data3$lable + 0.13)+

geom_segment(data = line, aes(x=x, y=y, xend= xend, yend=yend))+

labs(x = "", fill = "")+

theme_few(base_size = 10)+

theme(axis.text.x = element_text(angle = 45, hjust = 1,vjust = 1, colour = "black"),

legend.position = "top")

P

ggsave(paste0("fig.immu_cell_violin.pdf"),P,width = 10,height = 7)

Ssgsva$cluster <- group1$group

da <- data.frame(id=colnames(Ssgsva)[1:ncol(Ssgsva)-1])

rownames(da) <- da$id

for (i in da$id) {

da[i,2] <- wilcox.test(Ssgsva[,i] ~ Ssgsva$cluster)$p.value

}

da <- da[order(da$V2,decreasing = F),]

colnames(da) <- c("cell","wilcox.test.pvalue")

dat0.05 <- da[da$wilcox.test.pvalue < 0.05,] %>%

drop_na()

cell_exp <- (Ssgsva[,dat0.05$cell])

library(ggcor)

a2 <- quickcor(cell_exp,cor.test = TRUE,method = 'spearman') +

geom_square() +

scale_fill_gradient2(low = "#2b8cbe",mid = "white",high = "#e41a1c")+geom_mark(size = 5,r = NA)

ggsave("Fig.DEG_cell_cor_analysis.pdf",a2,width = 10,height = 8)

gene_expr <- as.data.frame(t(exper[myoverlap,]))

source("D:/xiangmu/stript/cor_heat.r")

cor_heat(gene_expr,cell_exp,outfile = "Fig.DEG_gene_cell")

data <- cbind(cell_exp,gene_expr)

library(ggpubr)

library(ggExtra)

for ( j in colnames(cell_exp)) {

for ( i in colnames(gene_expr)) {

df1=as.data.frame(data[,c(j,i)])

colnames(df1) <- c("y","x")

p1=ggplot(df1, aes(x, y)) +

xlab(i) + ylab(j)+

geom_point() + geom_smooth(method="lm",formula = y ~ x) + theme_bw()+

stat_cor(method = 'spearman', aes(x =x, y =y))

p2=ggMarginal(p1, type="density", xparams=list(fill = "orange"), yparams=list(fill = "blue"))

pdf(file=paste0(i,"_",j,"_cor.pdf"), width=5.2, height=5)

print(p2)

dev.off()

}

}

######

a <- correlate(cell_exp,gene_expr, cor.test = TRUE,method = 'spearman')

r <- as.data.frame(a$r)

write.csv(r,file = "gene_immu_cell_cor_R.csv")

p <- as.data.frame(a$p.value)

colnames(p) <- colnames(r)

rownames(p) <- rownames(r)

write.csv(p,file = "gene_immu_cell_cor_P.csv")

library(ggstatsplot)

for (i in 1:4) {

data <- data.frame(symbol= rownames(r),correlation=r[,i],pvalue=p[,i])

data %>%

#filter(pvalue <0.05) %>% # 如果不想把p值大于0.05的放在图上，去掉最前面的#号

ggplot(aes(correlation,forcats::fct_reorder(symbol,correlation))) +

geom_segment(aes(xend=0,yend=symbol)) +

geom_point(aes(col=pvalue,size=abs(correlation))) +

scale_colour_gradientn(colours=color2) +

scale_color_viridis_c(begin = 0.5, end = 1) +

scale_size_continuous(range =c(2,8)) +

theme_minimal() +

ggtitle(colnames(r)[i])+

ylab(NULL)

ggsave(paste0(colnames(r)[i],"_gene_immu_cell_cor.pdf"), width=8, height=8)

}

#############################09_immu_point_analysis######################################################

dir.create(paste0(Work_dir,"/09_immu_point_analysis"))

setwd(paste0(Work_dir,"/09_immu_point_analysis"))

load("GSE201332_DEG.Rdata")

imm_gene <-read.table("immunomodulator.txt",header = T,sep = "\t")

table(imm_gene$typle)

library(ggcor)

source("D:/xiangmu/stript/cor_heat.r")

gene_expr <- as.data.frame(t(exper[myoverlap,]))

##chemokine

chemokine <- imm_gene$gene[imm_gene$typle == "chemokine"]

chemokine_exp <- as.data.frame(t(exper[intersect(chemokine,rownames(exper)),]))

str(chemokine_exp)

cor_heat(chemokine_exp,gene_expr,outfile = "Fig.score_chemokine",width = 6,height = 8)

##Immunoinhibitor

Immunoinhibitor <- imm_gene$gene[imm_gene$typle == "Immunoinhibitor"]

Immunoinhibitor_exp <- as.data.frame(t(exper[intersect(Immunoinhibitor,rownames(exper)),]))

str(Immunoinhibitor_exp)

cor_heat(Immunoinhibitor_exp,gene_expr,outfile = "Fig.score_Immunoinhibitor",width = 6,height = 8)

##Immunostimulator

Immunostimulator <- imm_gene$gene[imm_gene$typle == "Immunostimulator"]

Immunostimulator_exp <- as.data.frame(t(exper[intersect(c(Immunostimulator,Immunoinhibitor,chemokine),rownames(exper)),]))

str(Immunostimulator_exp)

cor_heat(Immunostimulator_exp,gene_expr,outfile = "Fig.score_Immun_all",width = 6,height = 8)

######

a <- correlate(Immunostimulator_exp,gene_expr, cor.test = TRUE,method = 'spearman')

r <- as.data.frame(a$r)

p <- as.data.frame(a$p.value)

colnames(p) <- colnames(r)

rownames(p) <- rownames(r)

library(ggstatsplot)

for (i in 1:4) {

data <- data.frame(symbol= rownames(r),correlation=r[,i],pvalue=p[,i])

data %>%

#filter(pvalue <0.05) %>%

ggplot(aes(correlation,forcats::fct_reorder(symbol,correlation))) +

geom_segment(aes(xend=0,yend=symbol)) +

geom_point(aes(col=pvalue,size=abs(correlation))) +

scale_colour_gradientn(colours=color2) +

scale_color_viridis_c(begin = 0.5, end = 1) +

scale_size_continuous(range =c(2,8)) +

theme_minimal() +

ggtitle(colnames(r)[i])+

ylab(NULL)

ggsave(paste0(colnames(r)[i],"_gene_imm_gene_cor.pdf"), width=8, height=8)

}

#######################################10_ceRNA_analysis#############################################################

dir.create(paste0(Work_dir,"/09_score_gene_ceRNA_analysis"))

setwd(paste0(Work_dir,"/09_score_gene_ceRNA_analysis"))

#save(mirna_mrna_dabase,mirna_lncrna_dabase,circ_mirna_starbase,file = "starBase.Rdata")

myoverlap <- c("SLC25A5","ALDH2","CPT1C","IMMT")

#mrna

load("D:/xiangmu/starBase.Rdata")

mrna <- myoverlap

starbase <- mirna_mrna_dabase[mirna_mrna_dabase$geneName %in% mrna,]

starbase <- starbase[starbase$CancerNum > 0,]

mirWalk <- read.csv("miRWalk_miRNA_Targets (2).csv",header = T)

#mirWalk <- mirWalk[mirWalk$binding_region_length > 25,]

library(ggVennDiagram)

gene = list(MirWalk_mRNA_miRNA=mirWalk$mirnaid,

Starbase_mRNA_miRNA = starbase$name)

plot =ggVennDiagram(gene) + scale_fill_gradient(low="#D53E4F",high= "#3288BD")

plot

ggsave("MirWalk_Starbase_mrna_mirna_VEN.pdf",plot , width = 6, height = 6)

con_mirna <- intersect(starbase$name ,mirWalk$mirnaid)

write.csv(file = "con_mirna.csv",gsub("R","r",con_mirna))

mrna_mirna <- starbase[starbase$name %in% con_mirna,]

table(mrna_mirna$geneName)

Starbase_mirna_lncRNA <- mirna_lncrna_dabase[mirna_lncrna_dabase$miRNAname %in% con_mirna,]

Starbase_mirna_lncRNA <- Starbase_mirna_lncRNA[Starbase_mirna_lncRNA$pancancerNum > 7,]

unique(Starbase_mirna_lncRNA$geneName)

mirnet_mirna_lncRNA <- read.csv("mirnet_mir_target.csv",header = T)

mirnet_mirna_lncRNA$ID <- gsub("r","R",mirnet_mirna_lncRNA$ID)

gene = list(Starbase_mirna_lncRNA=Starbase_mirna_lncRNA$geneName,

mirnet_mirna_lncRNA = mirnet_mirna_lncRNA$Target)

plot =ggVennDiagram(gene) + scale_fill_gradient(low="#D53E4F",high= "#3288BD")

plot

ggsave("net_Starbase_lncrna_mirna_VEN.pdf",plot , width = 6, height = 6)

con_lnc <- intersect(Starbase_mirna_lncRNA$geneName,mirnet_mirna_lncRNA$Target)

mirna_lncRNA <- Starbase_mirna_lncRNA[Starbase_mirna_lncRNA$geneName %in% con_lnc,]

mrna_mirna1 <- mrna_mirna[mrna_mirna$name %in% intersect(mrna_mirna$name,mirna_lncRNA$miRNAname),]

unique(mrna_mirna1$geneName)

mirna_lncRNA1 <- mirna_lncRNA[mirna_lncRNA$miRNAname %in% intersect(mrna_mirna$name,mirna_lncRNA$miRNAname),]

unique(mirna_lncRNA1$miRNAname)

write.csv(file = "lncrna_mirna1.csv",mirna_lncRNA1)

write.csv(file = "mrna_mirna1.csv",mrna_mirna1)

#######################################10_score_gene_TF_analysis#############################################################

dir.create(paste0(Work_dir,"/10_score_gene_TF_analysis"))

setwd(paste0(Work_dir,"/10_score_gene_TF_analysis"))

#######################################11_score_gene_drug_analysis#############################################################

dir.create(paste0(Work_dir,"/11_score_gene_drug_analysis"))

setwd(paste0(Work_dir,"/11_score_gene_drug_analysis"))
