# Supplementary figures and images for: Investigating biomarkers of mitochondrial and aging-related genes in major depressive disorder through bioinformatics analysis
Source: Front Psychiatry. 2025 Sep 24;16:1653998. doi: 10.3389/fpsyt.2025.1653998 (PMC12504309; doi:10.3389/fpsyt.2025.1653998)

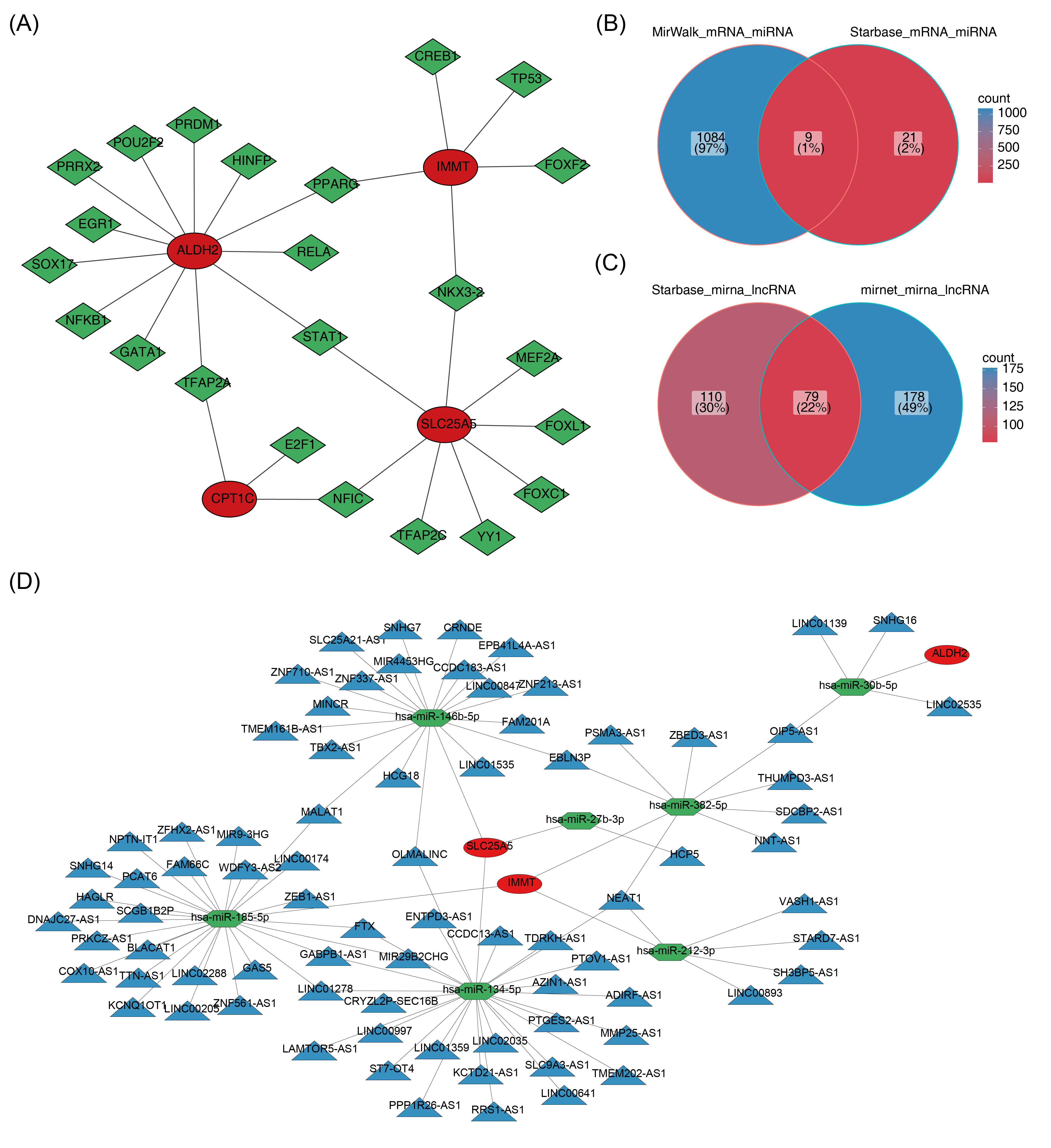

Supplement: Supplementary file 6 [file Image1.tif]
